# Abnormal triaging of misfolded proteins by adult neuronal ceroid lipofuscinosis-associated DNAJC5/CSPα mutants causes lipofuscin accumulation

**DOI:** 10.1080/15548627.2022.2065618

**Published:** 2022-05-04

**Authors:** Juhyung Lee, Yue Xu, Layla Saidi, Miao Xu, Konrad Zinsmaier, Yihong Ye

**Affiliations:** a Laboratory of Molecular Biology, National Institute of Diabetes and Digestive and Kidney Diseases, National Institutes of Health, Bethesda, MD, USA; b National Center for Advancing Translational Sciences, National Institutes of Health, Rockville, MD, USA; c Departments of Neuroscience and Molecular and Cellular Biology, University of Arizona, Tucson, AZ, USA

**Keywords:** DNAJC5/CSPα, cysteine string protein α, CLN4, ESCRT, lysobody, lysosome/endolysosome, microautophagy/eMI, misfolding-associated protein secretion/MAPS, neuronal ceroid lipofuscinosis/NCL, protein quality control/PQC, unconventional protein secretion/UPS

## Abstract

Mutations in *DNAJC5/CSPα* are associated with adult neuronal ceroid lipofuscinosis (ANCL), a dominant-inherited neurodegenerative disease featuring lysosome-derived autofluorescent *s*torage materials (AFSMs) termed lipofuscin. Functionally, DNAJC5 has been implicated in chaperoning synaptic proteins and in misfolding-associated protein secretion (MAPS), but how DNAJC5 dysfunction causes lipofuscinosis and neurodegeneration is unclear. Here we report two functionally distinct but coupled chaperoning activities of DNAJC5, which jointly regulate lysosomal homeostasis: While endolysosome-associated DNAJC5 promotes ESCRT-dependent microautophagy, a fraction of perinuclear and non-lysosomal DNAJC5 mediates MAPS. Functional proteomics identifies a previously unknown DNAJC5 interactor SLC3A2/CD98hc that is essential for the perinuclear DNAJC5 localization and MAPS but dispensable for microautophagy. Importantly, uncoupling these two processes, as seen in cells lacking SLC3A2 or expressing ANCL-associated DNAJC5 mutants, generates DNAJC5-containing AFSMs resembling NCL patient-derived lipofuscin and induces neurodegeneration in a *Drosophila* ANCL model. These findings suggest that MAPS safeguards microautophagy to avoid DNAJC5-associated lipofuscinosis and neurodegeneration.

**Abbreviations:** 3-MA: 3-methyladenine; ACTB: actin beta; AFSM: autofluorescent storage materials; ANCL: adult neuronal ceroid lipofuscinosis; Baf. A1: bafilomycin A_1_; CLN: ceroid lipofuscinosis neuronal; CLU: clusterin; CS: cysteine string domain of DNAJC5/CSPα; CUPS: compartment for unconventional protein secretion; DN: dominant negative; DNAJC5/CSPα: DnaJ heat shock protein family (Hsp40) member C5; eMI: endosomal microautophagy; ESCRT: endosomal sorting complex required for transport; GFP: green fluorescent protein; HSPA8/HSC70: heat shock protein family A (Hsp70) member 8; INCL: infant neuronal ceroid lipofuscinosis; JNCL: juvenile neuronal ceroid lipofuscinosis; KO: knockout; LAMP1: lysosomal associated membrane protein 1; LAPTM4B: lysosomal protein transmembrane 4 beta; LN: linker domain of DNAJC5/CSPα; MAPS: misfolding-associated protein secretion; mCh/Ch: mCherry; mCi/Ci: mCitrine; MTOR: mechanistic target of rapamycin kinase; NCL: neuronal ceroid lipofuscinosis; PPT1: palmitoyl-protein thioesterase 1; PQC: protein quality control; SBP: streptavidin binding protein; SGT: small glutamine-rich tetratricopeptide repeat; shRNA: short hairpin RNA; SLC3A2/CD98hc: solute carrier family 3 member 2; SNCA/α-synuclein: synuclein alpha; TMED10: transmembrane p24 trafficking protein 10; UV: ultraviolet; VPS4: vacuolar protein sorting 4 homolog; WT: wild type.

## Introduction

Neuronal ceroid lipofuscinosis (NCL) refers to a family of genetically inherited neurodegenerative lysosomal storage diseases that are associated with excessive accumulation of lipopigments (lipofuscin), which occurs in both neurons and non-neuronal tissues [1,2]. A key characteristic of lipofuscin is its autofluorescence, which allows detection by fluorescence microscopy [3]. These diseases can occur in either infants, juveniles, or adults. The infantile and juvenile variants of NCL (INCL and JNCL) are often more severe, associated with progressive vision loss, seizure, and brain death. The adult variant (ANCL also named CLN4) on the other hand has milder symptoms. Nevertheless, after diagnosis, ANCL patients usually die after 10 years [4].

Mutations in a collection of CLN genes have been linked to NCL diseases [4–6]. Many *CLN* genes encode proteins essential for lysosomal functions such as lysosomal proteases (e.g. CTSD) [4] or regulators governing the trafficking of lysosome resident proteins (e.g. CLN6 and CLN8) [7,8]. These observations suggested that lipofuscin accumulation and neurodegeneration in NCL might result from disrupted lysosome homeostasis.

ANCL is caused by dominant mutations in the *DNAJC5/CSPα* gene, which encodes a vesicle-associated chaperone protein. DNAJC5 features three conserved domains: an HSPA8/HSC70-binding J-domain near the N terminus, a central cysteine string (CS) domain, and a linker (LN) domain sandwiched between the J- and the CS-domain [9]. The cysteine residues in the CS domain are mostly palmitoylated, which regulate DNAJC5 membrane association and trafficking [10,11]. In neurons, DNAJC5 is mostly associated with synaptic vesicles [12–14] but a fraction of DNAJC5 is localized to endolysosomes [15]. In non-neuronal cells, DNAJC5 is mostly association with endolysosomes but the function of endolysosome-associated DNAJC5 is unclear [15–19]. As an HSPA8 co-chaperone, DNAJC5 can stimulate the ATPase activity of HSPA8 and HSPA/HSP70 in vitro [20,21]. In neurons, it can function in conjunction with these chaperones to control the folding and trafficking of synaptic proteins such as SNAP25 and DNM (dynamin) [22,23], which in turn regulate a variety of cellular processes including calcium homeostasis [24–27], membrane fusion [28,29], neurotransmitter release [29–31] and synapse stability [32–34]. Moreover, recent studies have also implicated DNAJC5 in unconventional secretion of misfolded cytosolic proteins via a process named misfolding-associated protein secretion (MAPS) [19,35,36]. Since the MAPS-promoting activity of DNAJC5 is tightly linked to its endolysosome association, it was proposed that DNAJC5 might chaperone misfolded proteins to the cell exterior via lysosomal exocytosis [35].

Two ANCL-associated mutations have been reported [37–39], which are located next to each other in the CS domain, close to the interface of the upstream LN domain whose function is unknown. Recent studies suggest that these mutations reduce DNAJC5 palmitoylation while increasing its aggregation propensity [15,40–42]. These mutations also cause mislocalization of the affected protein in cells [41]. Accordingly, the ANCL-causing *DNAJC5* mutations are thought to reduce the DNAJC5 chaperoning function [42]. However, this notion does not explain why cells carrying ANCL-associated *DNAJC5* mutations accumulate lipopigments in lysosomes.

In this study, we found that DNAJC5 used a J-domain independent activity to couple two protein quality control (PQC) processes: ESCRT-dependent endosomal microautophagy (eMI) and misfolding-associated protein secretion (MAPS). We identified SLC3A2/CD98hc as a DNAJC5 interactor that is critical for MAPS, but dispensable for microautophagy. Importantly, reducing SLC3A2-mediated protein secretion or expression of ACNL-associated DNAJC5 mutants uncouples these processes, resulting in lipofuscinosis and neurodegeneration.

## Results

### J-domain independent translocation of DNAJC5 into the lumen of endolysosomes

**Figure 1.**
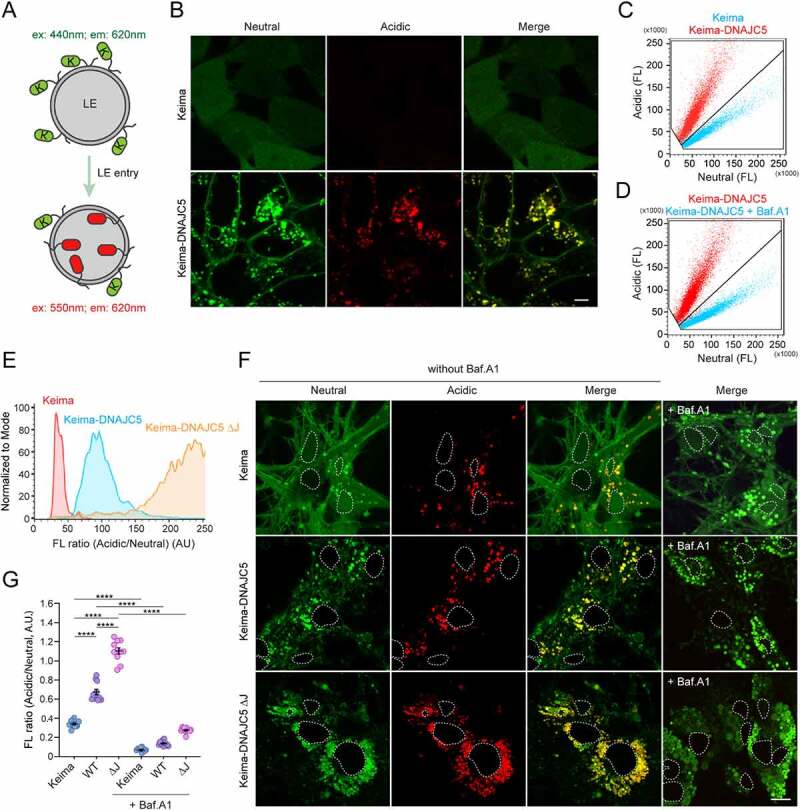
Translocation of DNAJC5 into the lumen of endolysosomes. (**A**) The Keima-based endolysosome translocation assay. (**B** and **C**) Keima-DNAJC5 but not Keima is efficiently translocated into endolysosomes. HEK293T cells stably expressing *Keima* or *Keima-DNAJC5* were analyzed by confocal microscopy using Ex_440_/Em_610_ (neutral) or Ex_550_/Em_610_ (acidic). (B) scale bar: 5 μm. (C) Representative Em_610_ population analysis of the Keima or Keima-DNAJC5 fluorescence intensity at Ex_440_ nm (neutral) and Ex_550_ nm (acidic) in stable cells using flow cytometry. (**D**) Flow cytometry analysis of Keima-DNAJC5 cells as (C) before and after bafilomycin A_1_ (200 nM, 2 h) treatment. FL, fluorescence. (**E**) Deletion of the J-domain stimulates DNAJC5 endolysosome translocation. The ratio of acidic vs. neutral fluorescence signals in cells stably expressing the indicated *Keima* proteins was analyzed by FACS. (**F** and **G**) Keima-DNAJC5 is translocated into endolysosomes in primary neurons. Mouse primary hippocampal neurons (DIV 10) expressing either *Keima, Keima-DNAJC5 WT* or *ΔJ* proteins were analyzed by confocal microscopy. The white dotted lines indicate nuclei. (G) shows the ratio of acidic vs. neutral fluorescence (mean intensity) in each image (*n* = 11 images from two independent experiments) measured by the Zeiss Zen (black) software. Note that treatment of bafilomycin A_1_ (100 nM, 6 h) abolished the acidic signals in neurons. Error bars indicate mean ± s.e.m. ****, *p* < 0.0001 by one-way ANOVA plus Tukey’s multiple comparisons test.

If DNAJC5 chaperones misfolded proteins for secretion using endolysosomes as a secretory compartment, DNAJC5 should accompany cargos to the lumen of endolysosomes. To test this idea, we established a Keima-based fluorescence assay. Keima is a monomeric dual-excitable fluorescence protein (λ^em^max ~620 nm) with maximum excitation at 440 nm under neutral pH (6.0–8.0) or at 550 nm in an acidic environment (pH<6.0) (Figure 1A) [43]. When fused to a cargo, Keima allows quantitative measurement of cargo trafficking to acidic endolysosomes by fluorescence microscopy or flow cytometry [44].

Confocal microscopy showed that HEK293T cells stably expressing *Keima* displayed only neutral cytoplasmic fluorescence. By contrast, in *Keima-DNAJC5* expressing cells, we not only detected neutral Keima-DNAJC5 signals on the plasma membrane but also observed Keima-DNAJC5 on intracellular vesicles by excitation at either 440 nm or 550 nm (Figure 1B). Thus, these vesicles are endolysosomes containing Keima-DNAJC5 both on the surface and in the lumen. Consistently, flow cytometry revealed an increased ratio of acidic to neutral Keima-DNAJC5 fluorescence when compared to Keima, and this phenotype was independent of the Kema-DNAJC5 expression level (Figure 1C). As anticipated, treatment with bafilomycin A_1_ (Baf. A1), a lysosome acidification blocker, dramatically reduced the acidic Keima-DNAJC5 signal while increasing the neutral Keima-DNAJC5 signal (Figure 1D). Thus, DNAJC5 can enter the lumen of endolysosomes.

To test if endosomal translocation of DNAJC5 requires the J-domain, we measured the ratio of the acidic vs. neutral fluorescence in cells stably expressing either *WT Keima-DNAJC5* or *a Keima-DNAJC5* mutant lacking the J-domain (*ΔJ*). Intriguingly, deleting the J-domain further enhanced the endolysosomal translocation of DNAJC5 (Figure 1E). This phenotype is likely achieved via a gain-of-function activity because the DNAJC5 ΔJ mutant was also more active in promoting MAPS (see below).

To confirm the endolysosomal translocation of DNAJC5 in neurons, we infected mouse primary neurons with lentiviruses expressing either *Keima, Keima-DNAJC5*, or *Keima-DNAJC5 ΔJ* under the *SYN1* (synapsin1) promoter on DIV 3 (Days in vitro). The *SYN1* promoter has been widely used to achieve neuronal specific gene expression at low levels. At DIV 10, we examined cells by confocal microscopy (Figure 1F). Interestingly, some acidic Keima punctae were observed in Keima-expressing neurons, probably due to macroautophagy/autophagy. Similar to non-neuronal cells, the acidic to neutral Keima ratio was increased in cells expressing *Keima-DNAJC5* compared to *Keima*-expressing cells. Furthermore, neurons expressing *Keima-DNAJC5 ΔJ* had the highest acidic to neutral Keima ratio (Figure 1F,G). As expected, Baf. A1 treatment diminished all acidic Keima signals (Figure 1F,G). Collectively, these results demonstrate the translocation of DNAJC5 into the lumen of endolysosomes in neurons.

### DNAJC5 promotes endolysosomal translocation of misfolded proteins via microautophagy

**Figure 2.**
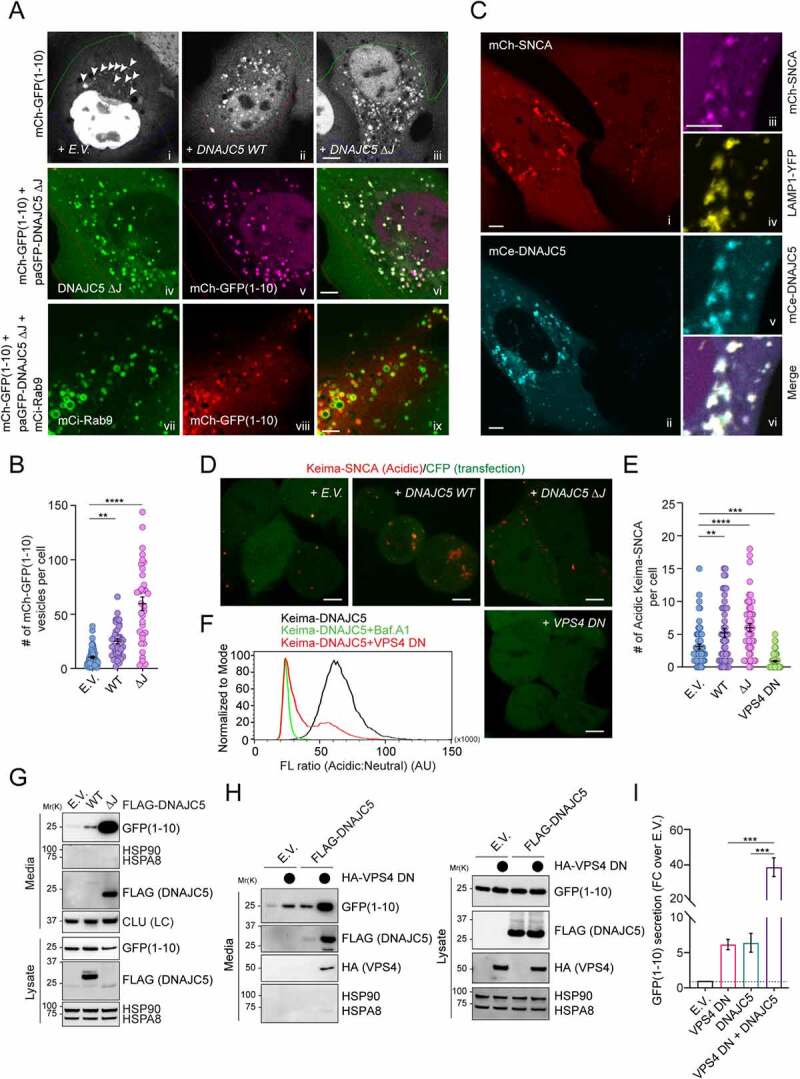
DNAJC5-mediated microautophagy is dispensable for MAPS. (**A-C**) DNAJC5 promotes the association of misfolded proteins with endolysosomes. (A) Panels i–iii, COS-7 cells transfected with *mCh-GFP1-10* together with either empty vector (EV) or *photoactivatable GFP (paGFP)-tagged DNAJC5 WT* or *DNAJC5 ΔJ* were subject to repeated photobleaching in the indicated areas, which reveals vesicle-associated mCh-GFP1-10 (arrowheads). The number of mCh-GFP1-10-containing vesicles in individual cells are shown in (B). Error bars indicate mean ± s.e.m. **, *p* < 0.005 (*p* = 0.0023); ****, *p* < 0.0001 by one-way ANOVA plus Dunnett’s multiple comparisons test; *n* = 50, 43, and 34 cells respectively from 3 independent experiments. Panels iv–vi show the colocalization of mCh-GFP1-10 with paGFP-DNAJC5 ΔJ. Panels vii–ix, cells transfected with *mCh-GFP1-10, DNAJC5 ΔJ*, and *mCi-Rab9* show the colocalization of mCh-GFP1-10 with RAB9. (C) U2OS cells stably expressing *mCh-SNCA* were transfected with *mCerulean (mCe)-DNAJC5* and *LAMP1-YFP* and imaged. The right panels show enlarged views from another cell. Scale bars: 5 μm. (**D** and **E**) DNAJC5 promotes the endolysosomal translocation of Keima-SNCA. *Keima-SNCA* stable HEK293T cells transfected with the indicated plasmids together with a *CFP*-expressing plasmid were imaged. CFP serves as a control for transfected cells. Scale bar: 5 μm. (E) shows the number of acidic Keima-SNCA-containing vesicles in individual cells in (D). Error bars indicate mean ± s.e.m. **, *p* < 0.005 (*p* = 0.0048); ***, *p* < 0.0005 (*p* = 0.0003); ****, *p* < 0.0001 by one-way ANOVA plus Dunnett’s multiple comparisons test. *n* = 63, 46, 48, and 77 cells respectively from 3 independent experiments. (**F**) VPS4 DN inhibits the endolysosomal translocation of Keima-DNAJC5. *Keima-DNAJC5* stable cells were treated with bafilomycin A_1_ or transfected with either an empty vector or *VPS4 DN^E228Q^*-expressing plasmid, and then analyzed by FACS. (**G-I**) DNAJC5-mediated secretion of misfolded proteins is independent of endolysosomal translocation. Conditioned medium and cell lysates from HEK293T cells transfected with *GFP1-10* together with the indicated plasmids were analyzed by immunoblotting. LC, loading control. (I) Quantification of the secreted GFP1-10 normalized by GFP1-10 in cell lysates. Error bars indicate mean ± s.e.m. of fold changes relative to E.V. control (dotted line), from *n* = 3 independent experiments. ***, *p* < 0.005 (*p* = 0.0002 for both) by one-way ANOVA plus Tukey’s multiple comparisons test.

To test whether DNAJC5 could chaperone misfolded proteins into endolysosomes, we used a truncated GFP protein (GFP1-10) tagged with mCherry (mCh-GFP1-10) as a model substrate. We previously developed a photobleaching-based imaging assay, which allowed detection of a small fraction of mCh-GFP1-10 that are associated with endolysosomes in live cells because photobleaching reduced the cytosolic mCh-GFP1-10 background [35]. Expression of *DNAJC5* increased the endolysosome association of mCh-GFP1-10, which was further enhanced in *DNAJC5 ΔJ*-expressing cells (Figure 2A, panels ii, iii vs. i; Figure 2B). As in *WT DNAJC5*-expressing cells, the mCh-GFP1-10-containing vesicles in *DNAJC5 ΔJ*-expressing cells also contain DNAJC5 ΔJ and the late endosomal protein RAB9 (Figure 2A, panels iv–ix). These results suggest that DNAJC5 recruits mCh-GFP1-10 to endolysosomes in a J-domain independent manner.

We performed a similar experiment using SNCA/α-synuclein (synuclein alpha), a misfolding-prone protein associated with Parkinson disease [45]. U2OS cells stably expressing *mCherry*-tagged human *SNCA* showed mainly a cytoplasmic mCh-SNCA signal, but a few SNCA-containing vesicles were detected without photobleaching (Figure 2C, panel i). *DNAJC5* expression significantly stimulated SNCA membrane association, resulting in many bright mCh-SNCA positive punctae (Figure 2C, panels i, ii). As expected, vesicle-associated SNCA was colocalized with DNAJC5 and the lysosomal marker LAMP1 (Figure 2C, panels iii–vi). Thus, DNAJC5 promotes the association of misfolded proteins with endolysosomes.

We next tested whether DNAJC5 could promote the translocation of misfolded proteins into the endolysosomal lumen using cells stably expressing *Keima-SNCA*, which is localized mostly in the cytoplasm with a few acidic Keima punctae, as demonstrated by confocal microscopy (Figure 2D). When *DNAJC5 WT* or *ΔJ* was co-expressed, however, the number of acidic Keima-SNCA punctae was significantly increased (Figure 2D,E).

Because microautophagy is a major mechanism that translocates proteins into the lumen of endolysosomes via ESCRT-dependent multivesicular body formation [46–49], we tested whether ESCRT-dependent microautophagy is involved in endolysosomal translocation of misfolded proteins by DNAJC5. We ectopically expressed a dominant-negative (DN) *VPS4 mutant^E228Q^*, which blocked ESCRT-mediated microautophagy [50]. As expected, the expression of VPS4 DN abolished acidic Keima-SNCA puncta (Figure 2D,E) and reduced the acidic Keima-DNAJC5 fluorescence as shown by flow cytometry (Figure 2F). LysoTracker staining showed that VPS4 DN did not affect the lysosomal pH (Figure S1A). Collectively, these results demonstrate that DNAJC5 chaperones misfolded proteins to the lumen of endolysosomes via ESCRT-dependent microautophagy.

### Endolysosomal translocation of misfolded proteins is dispensable for MAPS

We next asked whether DNAJC5-mediated microautophagy is required for MAPS. To this end, conditioned medium and cell lysate from HEK293T cells transfected with the MAPS substrate *GFP1-10* [35] were analyzed by immunoblotting, which detected a fraction of GFP1-10 but not abundant cytosolic proteins such as HSPA8 and HSP90 in the medium (Figure 2G and Figure S1B). Consistent with previous reports, co-expression of *DNAJC5* enhanced GFP1-10 secretion [19,36]. Strikingly, the MAPS-stimulating activity of DNAJC5 ΔJ was almost 10-fold higher than that of WT DNAJC5 (Figure 2G and Figure S1B). A similar observation was made for SNCA (Figure S1C). Notably, a fraction of WT DNAJC5 was also secreted, and the level of secreted DNAJC5 ΔJ was much higher than that of WT DNAJC5 (Figure 2G and Figure S1C). The correlation between endolysosomal translocation of DNAJC5 and its MAPS-stimulating activity appears to confirm endolysosomes as the secretory compartment for MAPS. However, when we examined the secretion of GFP1-10 in the presence of VPS4 DN, surprisingly, VPS4 DN dramatically increased GFP1-10 secretion under both basal and *DNAJC5*-overexpressing conditions (Figure 2H,I). These results suggest that DNAJC5-mediated microautophagy and MAPS are two parallel but functionally coupled quality control processes.

### ANCL mutations inhibit MAPS without affecting DNAJC5 endolysosomal translocation

**Figure 3.**
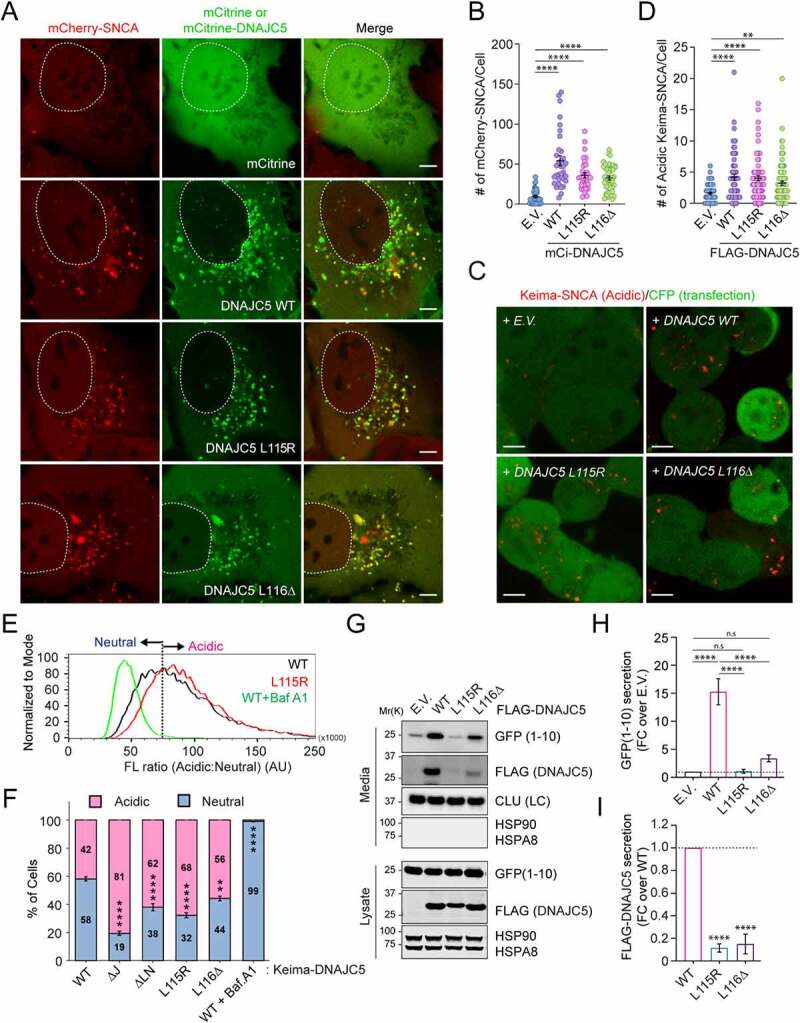
ANCL-associated DNAJC5 mutants are defective in MAPS but capable of translocating substrates into endolysosomes. (**A** and **B**) The DNAJC5^L115R^ and ^L116Δ^ mutants promote the association of mCh-SNCA with endolysosomes similarly as WT DNAJC5. Representative images of *mCh-SNCA*-expressing U2OS cells transfected with the indicated plasmids. Scale bar: 5 μm. (B) Quantification of mCh-SNCA-containing vesicles in individual cells in A. Error bars indicate mean ± s.e.m. ****, *p* < 0.0001 by one-way ANOVA plus Dunnett’s multiple comparisons test; *n* = 37, 33, 31, and 45 cells respectively from 3 independent experiments. (**C** and **D**) The DNAJC5^L115R^ and ^L116Δ^ mutants can still promote the translocation of Keima-SNCA into endolysosomes. (C) Representative images of Keima-SNCA cells transfected with the indicated plasmids together with *CFP*. Scale bar: 5 μm. (D) Quantification of mCh-SNCA-containing vesicles in individual cells in (C). Error bars indicate mean ± s.e.m. **, *p* < 0.005 (*p* = 0.0026); ****, *p* < 0.0001 by one-way ANOVA plus Dunnett’s multiple comparisons test; *n* = 81, 60, 69 and 88 cells respectively from 3 independent experiments. (**E** and **F**) The ANCL-associated DNAJC5 mutants are translocated into endolysosomal lumen more efficiently than WT DNAJC5. (E) Representative histograms show the ratio of acidic vs. neutral fluorescence intensity in cells transfected with the indicated plasmids. As a control, cells expressing *WT DNAJC5* were treated with bafilomycin A_1_ (200 nM) for 2 h. The line indicates the threshold by which cell populations were assigned and analyzed in (F). (F) Quantification of cell populations as indicated in (E) in cells transfected with the indicated plasmids. Error bars indicate mean ± s.e.m.; **, *p* < 0.005 (*p* = 0.0016); ****, *p* < 0.0001 by one-way ANOVA plus Dunnett’s multiple comparisons test; *n* = 3 experiments. (**G-I**) The ANCL-associated DNAJC5 mutants are defective in MAPS. Conditioned medium and cell lysates from cells transfected with *GFP1-10* together with the indicated plasmids were analyzed by immunoblotting. LC, loading control. The graph in (H) shows the quantification of the secreted GFP1-10 normalized by GFP1-10 in cell lysates. Error bars indicate mean ± s.e.m. of fold changes relative to E.V. control (dotted line), from *n* = 4 independent experiments. ****, *p* < 0.0001; n.s, not significant by one-way ANOVA plus Tukey’s multiple comparison test. The graph in (I) shows the secretion of DNAJC5 from *n* = 3 independent experiments. Error bars indicate mean ± s.e.m. of fold changes relative to WT (dotted line), from *n = 3* independent experiments. ****, *p* < 0.0001 by one-way ANOVA plus Dunnett’s multiple comparisons test.

To understand how the above-mentioned DNAJC5 functions were affected by ANCL-associated mutations, we first tested whether DNAJC5^L115R^ and ^L116Δ^ mutants still promote endolysosome association of misfolded SNCA using *mCh-SNCA*-expressing cells. Like WT DNAJC5, both mCitrine (mCi)-tagged DNAJC5^L115R^ and DNAJC5^L116Δ^ stimulated endolysosome association of mCh-SNCA (Figure 3A,B). Likewise, when the endolysosomal translocation of Keima-SNCA was analyzed, the disease-associated mutants were as active as WT DNAJC5 in promoting acidic Keima-SNCA positive puncta (Figure 3C,D). Importantly, when cells were transfected with Keima-tagged *WT DNAJC5* or the ANCL-associated mutants, both Keima-DNAJC5^L115R^ and ^L116Δ^ were translocated into endolysosomes more efficiently than WT DNAJC5 (Figure 3E,F). These results suggest that the ANCL mutations do not inhibit the microautophagy activity of DNAJC5.

We next examined whether these ANCL mutations alter the DNAJC5 function in MAPS. When the secretion of GFP1-10 was examined, unlike WT DNAJC5, both DNAJC5^L115R^ and DNAJC5^L116Δ^ failed to promote GFP1-10 secretion (Figure 3G,H). Likewise, these ANCL-associated DNAJC5 mutants also failed to induce the secretion of SNCA (Figure S1D). Additionally, compared to WT DNAJC5, the secretion of the DNAJC5^L115R^ and ^L116Δ^ mutants was also consistently reduced (Figure 3 G and I). Because these ANCL mutations significantly reduce DNAJC5 palmitoylation [42], and because deleting the CS domain or treating cells with a palmitoyltransferase inhibitor both inhibit the function of DNAJC5 in MAPS [19,51], it appears that DNAJC5 palmitoylation is essential for MAPS but dispensable for DNAJC5-mediated microautophagy. Importantly, our data exclude endolysosomes as a secretory intermediate compartment for MAPS, at least under conditions tested.

### A linker domain targets DNAJC5 to LAMP1-negative perinuclear vesicles

**Figure 4.**
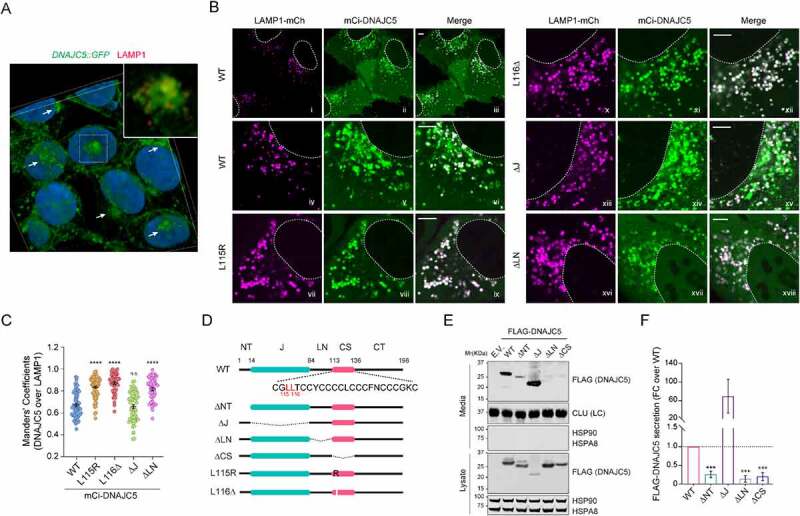
The linker domain localizes DNAJC5 to a perinuclear LAMP1-negative compartment and is required for MAPS. (**A**) A 3-D view of the subcellular localization of endogenous LAMP1 and DNAJC5 in HEK293T cells. HEK293T cells expressing *DNAJC5* bearing an endogenously tagged *GFP* at the C-terminus (*DNAJC5::GFP*) were stained with LAMP1 antibodies in red and DAPI in blue. Confocal images of z-stacks were reconstituted into a 3D view using the Nikon element software. The large image only shows the green and blue channel to highlight perinuclear DNAJC5 clusters (arrows). The inset is an enlarged view of the box-indicated area showing partially colocalization of DNAJC5-GFP with LAMP1. (**B** and **C**) DNAJC5 localization to a LAMP1-negative perinuclear compartment requires the LN domain. (B) Representative confocal images of U2OS cells transfected with *LAMP1-mCh* together with the indicated *mCitrine (mCi)-tagged DNAJC5* variants. Scale bars: 5 μm. (C) Quantification of the colocalization efficiency of various DNAJC5 variants with LAMP1-mCh in individual cells in B. Error bars indicate mean ± s.e.m. ****, *p* < 0.0001; n.s, not significant by one-way ANOVA plus Dunnett’s multiple comparisons test; *n* = 60, 66, 47, 60 and 45 cells respectively from 3 independent experiments. (**D**) A scheme illustrates the various DNAJC5 variants tested in B and E. (**E**) The LN domain is required for DNAJC5 secretion. Conditioned medium and cell lysates from HEK293T cells expressing the indicated *DNAJC5* variants were analyzed by immunoblotting. LC, loading control. (**F**) Quantification of the DNAJC5 secretion experiments shown in D. Error bars indicate mean ± s.e.m. of fold changes relative to WT (dotted line), from *n* = 3 independent experiments. ***, *p* < 0.0005 (*p* = 0.0006, 0.0002 and 0.0003, respectively) by one-way ANOVA plus Dunnett’s multiple comparisons test.

Having excluded endolysosome as a MAPS compartment, we re-characterized the subcellular localization of DNAJC5 using HEK293T cells bearing a GFP at the carboxyl terminus of endogenous DNAJC5 by 3D confocal microscopy (Figure S2A), which revealed a fraction of DNAJC5 in perinuclear vesicles largely negative for LAMP1 or the lysosome-specific dye LysoTracker (Figure 4A and Figure S2B). The perinuclear localization of DNAJC5 became more prominent in U2OS cells over-expressing *WT mCi-DNAJC5* (Figure 4B, panels i–vi). By contrast, the ANCL-associated mutants were largely absent from this compartment. As a result, both DNAJC5^L115R^ and ^L116Δ^ showed increased colocalization with LAMP1 (Figure 4B panels vii–xii).

To determine the domain(s) responsible for targeting DNAJC5 to the LAMP1 negative compartment, we characterized the localization of a set of DNAJC5 deletion mutants by confocal microscopy (Figure 4C,D). As previously reported [11], a DNAJC5 mutant lacking the CS domain (ΔCS) was entirely localized to the cytosol due to defects in both membrane binding and palmitoylation (Figure S2C,D). By contrast, Ci-DNAJC5 ΔNT (Figure S2C,D) and ΔJ (Figure 4B, panels xiii–xv; Figure 4C) were localized to both LAMP1-positive and LAMP1-negative vesicles similarly as WT DNAJC5, suggesting that HSPA8-binding is dispensable for DNAJC5 membrane association. Intriguingly, a Ci-DNAJC5 mutant lacking the linker (ΔLN) remained associated with LAMP1-positive vesicles but was largely absent from perinuclear vesicles (Figure 4B, panels xvi-xviii, Figure 4D). Keima-DNAJC5 ΔLN also showed increased endolysosomal translocation (Figure 3F). The similar localization of ΔLN and ANCL mutants (table S1) prompted us to test the secretion of DNAJC5 ΔLN by immunoblotting, which showed markedly reduced level of secretion similarly as DNAJC5 ΔCS (Figure 4E,F). These results suggest that the LN and CS domain act together to confer the perinuclear DNAJC5 localization, which is essential for DNAJC5-mediated MAPS.

### DNAJC5 interacts with SLC3A2 via the linker domain

**Figure 5.**
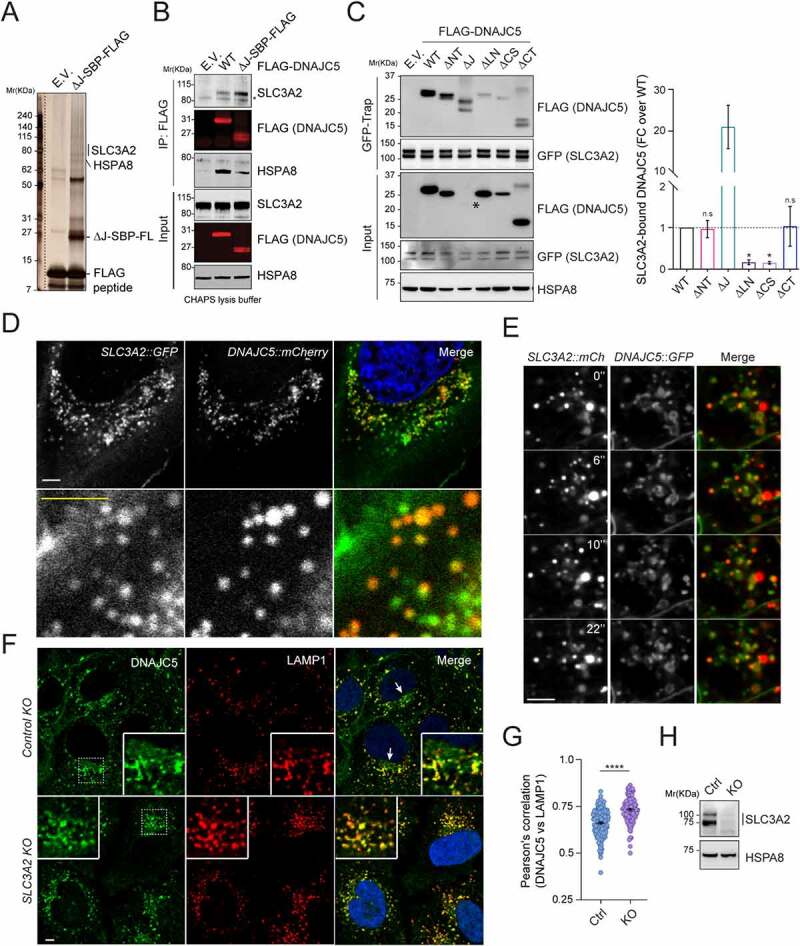
SLC3A2 interacts with DNAJC5 via the LN domain and is required for the perinuclear localization of DNAJC5. (**A**) A silver-stained gel showing the proteins co-purified with DNAJC5 ΔJ-SBP-FLAG. (**B**) Co-immunoprecipitation and immunoblotting confirm the interaction of both WT DNAJC5 and DNAJC5 ΔJ with endogenous SLC3A2. (**C**) The LN domain is required for DNAJC5 interaction with SLC3A2. *SLC3A2::GFP* cells were transfected with the indicated DNAJC5 variants for GFP-Trap and immunoblotting. The graph shows the quantification of SLC3A2-associated DNAJC5 normalized to DNAJC5 in cell lysates. Error bars indicate mean ± s.e.m. of fold changes relative to WT (dotted line). *, *p* < 0.05 (*p* = 0.0134 and 0.0446, respectively); n.s, not significant by one-way ANOVA plus Dunnett’s multiple comparisons test from at least 3 independent experiments. (**D** and **E**) Colocalization of endogenous DNAJC5 and SLC3A2. U2OS cells (in [D]) or HEK293 cells (in [E]) bearing GFP and mCherry tag on SLC3A2 and DNAJC5 respectively were imaged by dual-color confocal microscopy. (E) Representative frames from movie S1. Scale bars: 5 μm. (**F-H**) Depletion of *SLC3A2* abolishes a perinuclear pool of DNAJC5. (F) Representative images from control or *SLC3A2* knockout (KO) U2OS cells stained by DNAJC5 and LAMP1 antibodies. The arrows indicate perinuclear pool of DNAJC5. Scale bars: 5 μm. (G) Quantification of the colocalization efficiency of DNAJC5 and LAMP1 in (F). Error bars indicate mean ± s.e.m. ****, *p* < 0.0001 by one-way ANOVA plus Dunnett’s multiple comparisons test; *n* = 156 and 106 cells respectively from 3 independent experiments. (H) Immunoblotting confirms the depletion of *SLC3A2* in CRISPR KO cells.

To identify factor(s) that regulate the association of DNAJC5 with the LAMP1-negative compartment, we performed tandem affinity purification using cells stably expressing *DNAJC5 ΔJ* bearing FLAG- and SBP (Streptavidin binding protein) tags (Figure S3A,B). We used DNAJC5 ΔJ because of its strong MAPS-stimulating activity and also to avoid HSPA8 and its associated proteins. Mass spectrometry analyses identified SLC3A2/CD98hc, a cofactor of heterodimeric amino acid transporters [52], as a potential DNAJC5 binding partner (Figure 5A and Figure S3C,D). Additional co-immunoprecipitation showed that endogenous SLC3A2 interacts with not only DNAJC5 ΔJ but also WT DNAJC5, although WT DNAJC5 had a lower affinity to SLC3A2 than DNAJC5 ΔJ (Figure 5B). Reciprocal pulldown using a cell line bearing a GFP tag at the endogenous SLC3A2 locus further confirmed the interaction (Figure 5C and Figure S3E). Importantly, co-immunoprecipitation detected an interaction between endogenous DNAJC5 and SLC3A2 in mouse primary neurons (Figure S3F). Furthermore, when we transfected *DNAJC5 variants* into *SLC3A2::GFP* cells and performed GFP pulldown, DNAJC5 ΔLN and DNAJC5 ΔCS showed significantly reduced binding to SLC3A2 compared to other variants (Figure 5C).

To further confirm the interaction between SLC3A2 and DNAJC5, we examined the localization of *SLC3A2::GFP* by confocal microscopy. In addition to the plasma membrane, SLC3A2-GFP was detected on intracellular vesicles clustered around the nucleus (Figure S4A), a pattern similar to that of endogenous DNAJC5. Like DNAJC5, SLC3A2-GFP was localized to LAMP1- and LysoTracker-positive vesicles, but many SLC3A2-positive signals around the nucleus were free of LAMP1 (Figure S4B to B”). As anticipated, cells expressing GFP and mCherry on endogenous DNAJC5 and SLC3A2, respectively, showed extensive colocalization of these proteins on vesicles (Figure 5D,E; movie S1). Altogether, these results demonstrate a specific interaction between SLC3A2 and DNAJC5, which requires the DNAJC5 linker (LN) and CS domains.

### SLC3A2 is required for the perinuclear association of DNAJC5 and MAPS

The observation that DNAJC5 ΔLN, defective in SLC3A2 interaction, is also absent from the LAMP1-negative perinuclear compartment (Figure 4B) suggests SLC3A2 as a potential regulator of DNAJC5 localization. To test this idea, we immunostained endogenous DNAJC5 and LAMP1 in SLC3A2-depleted or control knockout cells. In control cells, the presence of DNAJC5 in the LAMP1 negative compartment was readily visible. By contrast, in SLC3A2 KO cells, DNAJC5 was almost completely absent from this compartment (Figure 5F–H; Figure S5A,B), while its colocalization with LAMP1 became more prominent. By contrast, the plasma membrane-associated DNAJC5 was not affected in *SLC3A2*-depleted cells (Figure S5A,B). Flow cytometry showed that *SLC3A2* deficiency or overexpression did not significantly affect the endolysosomal translocation of Keima-DNAJC5 (Figure S5C,D). Thus, SLC3A2 is specifically required for the perinuclear association of DNAJC5 but dispensable for its endolysosomal translocation.

**Figure 6.**
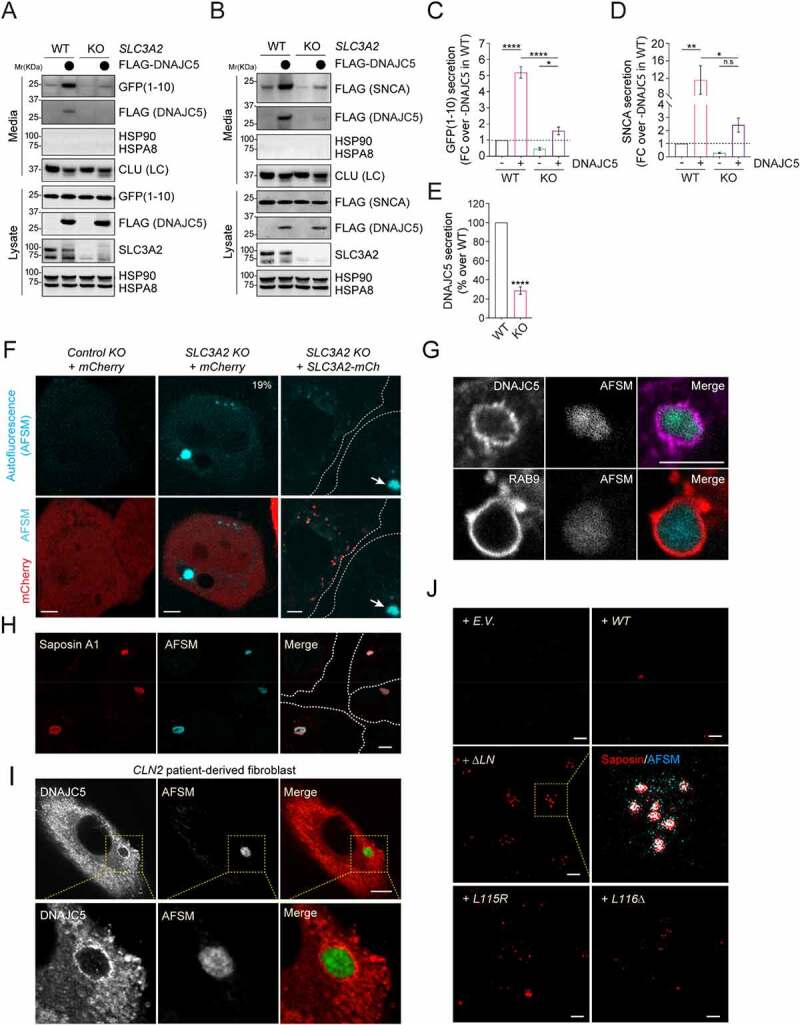
Depletion of *SLC3A2* inhibits MAPS and causes the accumulation of AFSMs in cells. (**A**) Depletion of *SLC3A2* inhibits GFP1-10 secretion. Conditioned medium and cell lysates from the control or *SLC3A2 KO* cells transfected with *GFP1-10* and the indicated plasmids were analyzed by immunoblotting. (**B**) As in A, except that cells transfected with *FLAG-SNCA* and the indicated plasmids were analyzed. (**C**) Quantification of the experiments in A (*n* = 4). Error bars indicate mean ± s.e.m. of fold changes relative to E.V control in *SLC3A2 WT* cell (dotted line). ****, *p* < 0.0001; *, *p* < 0.05 (p = 0.017) by Tukey’s multiple comparisons test. (**D**) Quantification of the experiments in (B) (*n* = 3). Error bars indicate mean ± s.e.m. of fold changes as seen in (C). **, *p* < 0.01 (*p* = 0.0082); *, *p* < 0.05 (*p* = 0.0183); n.s, not significant by Tukey’s multiple comparisons test. (**E**) Quantification of the DNAJC5 secretion from the experiments in (A) and (B). Error bars indicate mean ± s.e.m. of fold changes relative to *SLC3A2 WT cell*, from *n* = 4 independent experiments. ****, *p* < 0.0001 by one-tailed t-test. (**F-J**) *SLC3A2* deficient cells contain AFSMs resembling NCL-associated lipofuscin. (F) Control (Ctrl.) KO or *SLC3A2 KO* HEK293T cells were transfected with *mCherry (mCh)* or *SLC3A2-mCh* and imaged by confocal microscopy. The dashed lines indicate cell boundary. Arrow indicates a “lysobody” in a cell without SLC3A2-mCh. (G) Enlarged images of *SLC3A2 KO* cells stained by either DNAJC5 antibodies (Top panels) or transfected with *mCh-Rab9* (Bottom panels) show that AFSMs are surrounded by DNAJC5- and RAB9-positive membranes. (H) *SLC3A2 KO* U2OS cells stained with saposin A1 antibodies in red. (I) Fibroblast cells from NCL2 patients were stained with DNAJC5 antibodies. (J) HEK293T cells transfected with the indicated *DNAJC5* variants were stained by saposin A1 antibodies in red. Scale bars: 5 μm.

We next tested whether SLC3A2 is required for DNAJC5-mediated MAPS using SNCA and GFP1-10 as model substrates. Indeed, the secretion of these proteins under basal conditions or in *DNAJC5*-overexpressing cells was significantly reduced when *SLC3A2* was knocked down by 60–80% in HEK293T cells (Figure 6A–D; Figure S5E–G). Moreover, the secretion of DNAJC5 itself was also reproducibly inhibited in *SLC3A2* deficient cells (Figure 6A,B and E). The secretion of SNCA expressed under the *SYN1* promoter from primary neurons was also reduced by *SLC3A2* knockdown (Figure S5H,I; Figure S6D). These results suggest a role for SLC3A2 in DNAJC5-mediated MAPS.

The link of the perinuclear DNAJC5 to unconventional protein secretion prompted us to investigate whether DNAJC5 is colocalized with TMED10, a recently identified unconventional protein secretion regulator that is localized to ERGIC vesicles [53]. We endogenously tagged DNAJC5 with GFP and TMED10 with mCherry. Dual-color fluorescence microscopy showed that the perinuclear DNAJC5 is not colocalized with TMED10 in U2OS cells (Figure S5J).

### Depletion of SLC3A2 causes lipofuscin-like structures in mammalian cells

Given the link between DNAJC5 and lipofuscinosis, we determined whether *SLC3A2* deficiency caused accumulation of lipofuscin-like autofluorescent storage materials (AFSMs). Indeed, confocal microscopy analyses of more than 200 cells showed that ~19% of *SLC3A2* KO HEK293T cells contained one or two large spherical puncta detectable by excitation with a UV light (Figure 6F and Figure S6A). By contrast, AFSMs were only detected in less than 0.5% of WT cells. Importantly, AFSMs were barely detected when *SLC3A2* was re-expressed in the KO cells, suggesting that the AFSM accumulation was caused by *SLC3A2* depletion (Figure 6F). Knockdown of *Slc3a2* in mouse primary neurons also caused AFSM accumulation (Figure S6B–D). 3D confocal analysis combined with dual-color fluorescence microscopy showed that AFSMs in *SLC3A2* knockout cells were surrounded by membranes enriched for DNAJC5 and RAB9, consistent with an origin from endolysosomes (Figure 6G and Figure S6E). Additional immunostaining detected Saposin A1, a known marker of lipofuscin [54] in all AFSMs observed in *SLC3A2* KO cells (Figure 6H), confirming their identity as lipofuscin. Additionally, cells derived from late INCL patients bearing *CLN2* mutations also contained large lipofuscin-like AFSMs that were wrapped by DNAJC5-positive membranes (Figure 6I) and morphologically indistinguishable from those in *SLC3A2* KO cells [55]. Interestingly, cells overexpressing *DNAJC5 ΔLN, L115R* or *L116Δ* also contained an increased number of Saposin A1-positive AFSMs (Figure 6J), although they were smaller in size compared to those in *SLC3A2* KO cells, possibly because of the short expression time. Why knockdown of *SLC3A2* only generates AMSFs in a fraction of the cells remains to be elucidated. However, because *CLN2* encodes a lysosomal peptidase, our findings suggest that lipofuscin biogenesis is associated with either defective lysosomal degradation or excessive flow of proteins and membranes into endolysosomes (see discussion).

### AFSM accumulation and SLC3A2 deficiency contribute to neurodegeneration in a fly ANCL model

**Figure 7.**
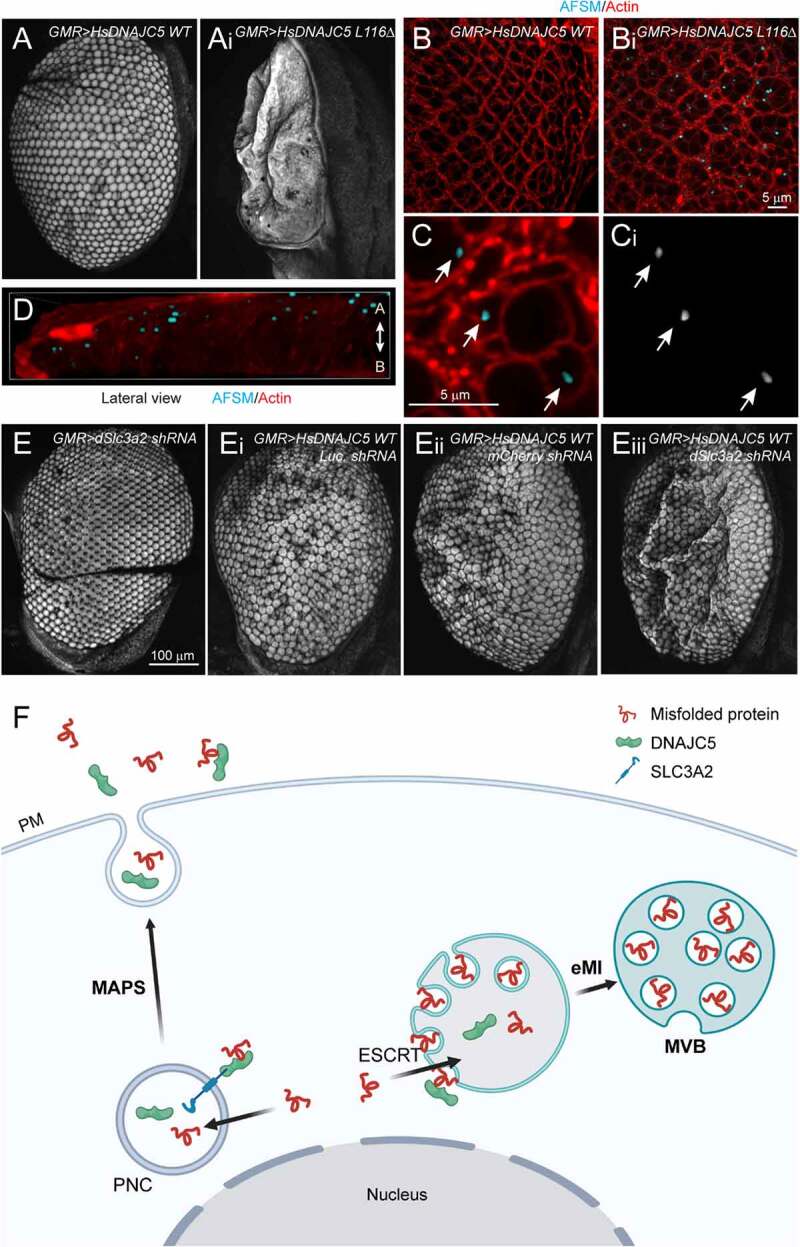
AFSM accumulation and genetic interaction between DNAJC5 and SLC3A2 in a fly ANCL model. (**A** and **Ai**) The expression of human *DNAJC5^L116Δ^* but not *DNAJC5 WT* in fly photoreceptor cells causes a severe rough eye phenotype. (**B-D**) AFSM accumulation in photoreceptor cells in larvae expressing human *DNAJC5^L116Δ^*. (B-Bi) Imaginal eye discs from *GMR>HsDNAJC5 WT* or *GMR>HsDNAJC5^L116Δ^* were stained with phalloidin to label cortex actin (red) and imaged at Ex_405_ to detect AFSM. (C-Ci) An enlarged view of a cluster of photoreceptor cells bearing AFSMs (arrows) in the cytoplasm. (D) A 3D side view of AFSM in a *DNAJC5^L116Δ^*-expressing eye disc. (A) apical; (B) Basal. (**E-Eiii**) d*Slc3a2* knockdown enhances the rough eye phenotype associated with *HsDNAJC5 WT* expression at 28°C. (**F**) The DNAJC5-mediated protein triaging pathways, and the deregulation of these processes in cells expressing ANCL-associated *DNAJC5* mutants. The illustration was created by Biorender. PM, plasma membrane; PNC, perinuclear compartments; eMI, endosomal microautophagy; MVB, multivesicular bodies.

We used a recently established fly model to further evaluate the role of DNAJC5 and SLC3A2 in neurodegeneration. We expressed *WT human DNAJC5 (HsDNAJC5)* or the ANCL-associated *DNAJC5^L116Δ^* mutant in photoreceptor cells of *Drosophila* larval eyes using the *GMR-Gal4* driver. As reported previously [41], *DNAJC5^L116Δ^* overexpression caused massive neuronal cell death, resulting in a severe rough eye phenotype in adult flies raised at 25°C (Figure 7A). By contrast, WT DNAJC5 did not change the eye morphology (Figure 7A). Interestingly, confocal microscopy analyses of larval eye discs showed that *DNAJC5^L116Δ^*-expressing tissues contained many autofluorescent punctae in apical cytoplasm within photoreceptor cells (Figure 7B–D; Figure S7A).

Our data suggests that SLC3A2 regulates the DNAJC5 localization and MAPS. If disruption of MAPS while maintaining DNAJC5-mediated microautophagy contributes to AFSM production and neurodegeneration, knockdown of *SLC3A2* should enhance DNAJC5-induced neurodegeneration. We, therefore, crossed *GMR>HsDNAJC5* WT and *GMR>HsDNAJC5*^L116Δ^ flies to a strain bearing a *SLC3A2*-targeting shRNA downstream of the UAS regulatory element, or as controls to strains bearing either a *luciferase shRNA* or *mCherry shRNA*. Expression of *SLC3A2 shRNA* resulted in ~60% depletion of endogenous *SLC3A2* mRNA (Figure S7B). When raised at 28°C, flies expressing *WT HsDNAJC5* together with *a control shRNA* suffered a modest rough eye phenotype, but no significant pigment loss (Figure 7E’ and E”; Figure S7C,D). By contrast, although flies expressing *SLC3A2 shRNA* alone had normal eyes (Figure 7E and Figure S7E), flies expressing *WT HsDNAJC5* together with *SLC3A2 shRNA* had more severe rough eyes (Figure 7E”’) with a significant loss of pigments (Figure S7C,D). Likewise, knockdown of *SLC3A2* also enhanced the rough eye phenotype in *hDNAJC5^L116Δ^*-expressing flies raised at 25°C (Figure S7E,F). Thus, depletion of *SLC3A2* enhances neuronal cell death induced by *DNAJC5* overexpression, probably because it disrupts the balance between DNAJC5-mediated MAPS and microautophagy, causing a disproportionally increase in the flow of misfolded proteins and membranes to endolysosomes.

## Discussion

Our study fails to establish endolysosome as an unconventional secretion compartment for MAPS. Instead, we find that DNAJC5-mediated MAPS and eMI are two tightly coupled PQC mechanisms essential for endolysosomal homeostasis. ANCL-associated *DNAJC5* mutations abolish DNAJC5 function in MAPS but maintain its microautophagy-stimulating activity (Figure 7F), causing lipofuscinosis and neurodegeneration. Specifically, we show that a fraction of DNAJC5 can be efficiently translocated into endolysosomes together with its clients. It was reported previously that selective eMI is mediated by HSPA8 and ESCRT proteins [47,48,50]. Although DNAJC5-mediated microautophagy is not dependent on its HSPA8-interacting J-domain, whether HSPA8 or other DNAJC5-associated chaperones such as SGT [14] function in substrate recruitment in this process remains to be tested. Intriguingly, recent studies showed that ANCL-associated mutations reduce DNAJC5 palmitoylation [42]. These mutants are also more prone to aggregation [40,41,56]. Nevertheless, these mutants are more efficiently translocated into endolysosomes compared to WT DNAJC5, suggesting that DNAJC5 palmitoylation is not essential for microautophagy. Indeed, it is known that DNAJC5 palmitoylation defective mutants can still bind to membranes in cells [11].

A fraction of DNAJC5 is also localized to a perinuclear membrane compartment, which is largely free of LAMP1 and not stained well by a LysoTracker dye. However, this compartment can be weakly labeled by LysoTracker after prolonged staining (movie S2), suggesting that it might be a pre-lysosomal compartment. The localization of DNAJC5 to this compartment does not require its J-domain but depends on palmitoylation of DNAJC5 since both the ANCL-associated disease mutants (this study) and a CS-domain deleted mutant fail to associate with this compartment [19]. Additionally, we identified a DNAJC5 binding partner named SLC3A2, which regulates the peri-nuclear localization of DNAJC5. SLC3A2 is a type II membrane glycoprotein capable of interacting with six light chain molecules, forming a set of amino acid transporters on the plasma membrane [52]. Moreover, additional adaptors such as LAPTM4B can retain a SLC3A2-containing transporter in endolysosomes, regulating the amino acid balance between endolysosome and cytoplasm and thus the MTOR signaling [57]. Whether SLC3A2-dependent regulation of the DNAJC5 localization requires additional components awaits further characterization.

Although the DNAJC5-associated pre-lysosomal compartment does not overlap with TMED10, a cis-Golgi protein recently implicated in unconventional protein secretion, several lines of evidence suggest that this compartment functions in MAPS. First, DNAJC5 mutants defective in binding to this compartment all fail to promote MAPS. Additionally, knockout of SLC3A2 impairs the association of DNAJC5 to this compartment, which also reduces the secretion of misfolded proteins. In *S. Cerevisiae*, unconventional protein secretion under stress conditions is mediated by a Golgi-derived compartment termed CUPS [58,59]. The pre-lysosomal DNAJC5-positive compartment may be functionally analogous to CUPS. In yeast, protein translocation into CUPS is thought to be mediated by the ESCRT machinery and some autophagy regulators [59]. By contrast, unconventional secretion of misfolded proteins in mammalian cells is not dependent on ESCRT and cannot be blocked by the autophagy inhibitor 3-MA [35]. Noticeably, previous studies have suggested a role for HSPA8 in USP19-stimulated MAPS [19,36]. The fact that neither the peri-nuclear localization of DNAJC5 nor its MAPS-stimulation activity depends on the J-domain raises the possibility that HSPA8 may collaborate with USP19 directly during substrate recruitment considering the reported interaction of USP19 with HSPA8 [60]. How misfolded proteins enter the DNAJC5-associated perinuclear compartment and the role of HSPA8 in this process remainto be elucidated.

DNAJC5-mediated microautophagy and MAPS appear to operate in parallel to maintain protein homeostasis. Conceivably, a potential benefit from these coupled PQC processes is to prevent overflow of toxic materials to endolysosomes. These processes, when properly tuned, should reduce misfolded proteins and improve cell homeostasis. By contrast, deregulation of these processes may lead to the accumulation of misfolded proteins in either endolysosomes or the cell exterior. The fact that DNAJC5 lacking the J-domain has significantly increased activities in both endolysosomal translocation and protein secretion suggests an autoinhibitory mechanism that tightly controls these processes. Consistently, structural studies have illustrated phosphorylation-dependent conformational changes, which disrupt a J-domain-mediated intermolecular interaction [61].

Our findings suggest that ANCL-associated *DNAJC5* mutations cannot be classified as simple loss- or gain-of-function alleles. Instead, while these mutations abolish the pre-lysosomal localization of DNAJC5 and the corresponding MAPS function, they increase the translocation of DNAJC5 into lysosomes. One presumed consequence is the abnormal flow of misfolded proteins and membranes into endolysosomes, damaging this organelle over time and resulting in undigested remnants in the form of lipofuscin. This model is consistent with the finding that mutations in PPT1 (palmitoyl protein thioesterase 1) also result in similar disease phenotypes [62]. Presumably, endolysosome-associated DNAJC5 is processed by PPT1, which may regulate its function in proteostasis regulation. Additionally, recent studies showed that ANCL-associated DNAJC5 mutants, albeit lacking MAPS activity, can still promote exosome biogenesis [51,63], further consolidating the proposed link between gain of DNAJC5 function in eMI and ANCL.

## Materials and methods

### Cell lines, siRNAs, plasmids, and antibodies

HEK293T, HEK293, COS-7, and U2OS cells were purchased from ATCC (ACS-4500, CRL-1573, CRL-1651, and HTB-96). Human patient derived *CLN2* fibroblast cell is a gift from Wei Zhang (NCATS/NIH). The cells were maintained in DMEM medium (Corning cellgro, 10–013-CV) supplemented with 10% fetal bovine serum (Gibco, 16,140,071) and penicillin-streptomycin (Gibco, 15,140,122). Transfection was performed with TransIT-293 (Mirus, MIR 2704) for HEK293T cells, and with Lipofectamine 2000 (Invitrogen, 11,668,030) for COS-7 and U2OS cells. Lipofectamine RNAiMAX (Invitrogen, 13,778,030) was used in all gene silencing experiments according to the manufacturer<apos;>s protocol. HEK293T and U2OS cells were used to stably express or were depleted of target proteins by lentivirus infection and selection for 3–7 days, using puromycin (0.3 μg/mL for HEK293T, 1 μg/mL for U2OS) or hygromycin (200 μg/mL). To generate *SLC3A2* CRISPR knockout HEK293T cell lines, we constructed two sgRNA-CAS9 guide constructs in pX330 hCas9 D10A (a gift from Feng Zhang; Addgene, 42,335) based on a published protocol [64]. The primers containing targeting sequences for the human *SLC3A2* gene are:

Target 1, forward primer: 5’-caccGCTGCAGATCGACCCCAATTT-3’;

Target 1, reverse primer: 5’-aaacAAATTGGGGTCGATCTGCAGc-3’;

Target 2, forward primer: 5’-caccGTCTGAGCGACATCATCCTTC-3’;

Target 2, reverse primer: 5’-aaacGAAGGATGATGTCGCTCAGAc-3’

The two *SLC3A2* targeting constructs were co-transfected into HEK293T cells. At 24 h post-transfection, cells were diluted and seeded into a 96-well plate at <1 cell per well. Single cell-derived clones were screened for *SLC3A2* deficiency by immunoblotting. To generate *SLC3A2* CRISPR knockout U2OS cell line, a sgRNA guide sequence (the Target 1 sequence above) was cloned into pLenti CRISPRv2 vector, and the lentivirus was used for infection as described previously [65].

Endogenous tagging was performed as described previously [66] (http://www.pcr-tagging.com). Briefly, the PCR cassettes were amplified from pMaCTag-05 plasmid (a gift from Michael Knop; Addgene, 119,984) by the AccuPrime™ Pfx DNA polymerase (Invitrogen, 12,344) using the primers listed below:

M1_*DNAJC5*, forward primer: 5’-ACGCCGATCGTCATACAGCCGGCATCCGCCACCGAGACCACCCAGCTCACAGCCGACTCCCACCCCAGCTACCACACTGACGGGTTCAACTCAGGTGGAGGAGGTAGTG-3’

M2_*DNAJC5*_enAsCas12a, reverse primer: 5’-TAAGGTTGGCGTGGCCAGGCGCCGGCTCCTCCTCTGACCACAGCTCCTCCTGGATAAAAAAAGCTCCTCCTGGATTTAGTTATCTACAAGAGTAGAAATTAGCTAGCTGCATCGGTACC-3’

M1_*SLC3A2*, forward primer: 5’-CCAGGCCGTGAGGAGGGCTCCCCTCTTGAGCTGGAACGCCTGAAACTGGAGCCTCACGAAGGGCTGCTGCTCCGCTTCCCCTACGCGGCCTCAGGTGGAGGAGGTAGTG-3’

M2_*SLC3A2*_enAsCas12a, reverse primer: 5’-GCCAAAGGGCCTGGGAAGGAAAGGAGAAGGGTAGTGGGTCCATGTCAGGCTGAAGAAAAAATGAAGTCAGGCCGCGTAGGGATCTACAAGAGTAGAAATTAGCTAGCTGCATCGGTACC-3’

M1_*TMED10*, forward primer: 5’-AGCATCTTTTCAATGTTCTGTCTCATTGGACTAGCTACCTGGCAGGTCTTCTACCTGCGACGCTTCTTCAAGGCCAAGAAATTGATTGAGTCAGGTGGAGGAGGTAGTG-3’

M2_*TMED10*_enAsCas12a, reverse primer: 5’-CCAGCGATGTTCTGCTGGCTGAGGTACAAGGTGGGAGGAGAATATGCCTCATTCAAAAAAATGCCTCATTCATTACTCAATATCTACAAGAGTAGAAATTAGCTAGCTGCATCGGTACC-3’

The PCR products were gel purified using a QIAGEN Gel Extraction Kit. HEK293T cells were transiently transfected with 1 µg of a PCR cassette and 1 µg of pCAG-enAsCas12a(E174R/S542R/K548R)-NLS(nuc)-3xHA (a gift from Keith Joung & Benjamin Kleinstiver; Addgene, 107,941) using TransIT293 (Mirus) according to the manufacturer<apos;>s protocol and GFP- or mCherry-positive cells were sorted two weeks later by FACS. For making double-tagging cells (*DNAJC5::GFP SLC3A2::mCherry*), The PCR cassette for *SLC3A2* described above was transfected to *DNAJC5::GFP* cell and cells showing double fluorescences were sorted by FACS. Endogenous tagged cell lines used in this study were all validated by immunoblottings (Figure S2A and Figure S3E). All expression constructs, si-RNAs, chemicals, and antibodies are listed in Table 1.

**Table 1.** Table with information about plasmids, siRNAs, chemicals, and antibodies.

Reagents	Source	Identifier
**Recombinant DNAs**		
SLC3A2/CD98hc-mCherry	This study	
SLC3A2/CD98hc-MYC-FLAG	Origene	
EGFP C1-SNCA/α-Synuclein	David Rubinsztein	Addgene, 40,822
EGFP-C1-VPS4 E228Q (DN)	Wesley Sundquist	Addgene, 80,351
FSW EGFP-SNCA/α-Synuclein	This study	
FSW mCherry	This study	
GFP(1–10)	Sandia Biotech	
LAMP1-pEYFP-N1	Walther Mothes	Addgene, 1816
mCerulean C1		
mCerulean C1-DNAJC5/CSPα	This study	
mCherry C1		
mCherry C1-GFP(1–10)		[35]
mCherry C1-RAB9Aa		[35]
mCherry C1-SNCA/α-Synuclein	This study	
mCitrine C1		
mCitrine C1-DNAJC5/CSPα L115R	This study	
mCitrine C1-DNAJC5/CSPα L116Δ	This study	
mCitrine C1-DNAJC5/CSPα WT	This study	
mCitrine C1-DNAJC5/CSPα ΔCS	This study	
mCitrine C1-DNAJC5/CSPα ΔJ	This study	
mCitrine C1-DNAJC5/CSPα ΔLN	This study	
mCitrine C1-RAB9A		[35]
mKeima Red C1	Michael Davidson	Addgene, 54,546
mKeima Red C1-DNAJC5/CSPα L115R	This study	
mKeima Red C1-DNAJC5/CSPα L116Δ	This study	
mKeima Red C1-DNAJC5/CSPα WT	This study	
mKeima Red C1-DNAJC5/CSPα ΔJ	This study	
mKeima Red C1-DNAJC5/CSPα ΔLN	This study	
mKeima Red C1-SNCA/α-Synuclein	This study	
pcDNA3-CSPα ΔJ-SBP-FLAG	This study	
pcDNA3-HA-VPS4 E228Q (DN)	This study	
pCMV6 entry	Origene	
pCMV6 entry-SNCA/α-Synuclein-MYC-FLAG	Origene	
pCMV6-XL6-FLAG-DNAJC5/CSPα L115R	This study	
pCMV6-XL6-FLAG-DNAJC5/CSPα L116Δ	This study	
pCMV6-XL6-FLAG-DNAJC5/CSPα WT	Chad A Dickey (University of South Florida)	
pCMV6-XL6-FLAG-DNAJC5/CSPα ΔCS	This study	
pCMV6-XL6-FLAG-DNAJC5/CSPα ΔCT	This study	
pCMV6-XL6-FLAG-DNAJC5/CSPα ΔJ	This study	
pCMV6-XL6-FLAG- DNAJC5/CSPα ΔLN	This study	
pCMV6-XL6-FLAG- DNAJC5/CSPα ΔNT	This study	
pLAMP1-mCherry	Amy Palmer	Addgene, 45,147
pLenti CMV hygro	This study	
pLenti CMV hygro-FLAG-DNAJC5/CSPα L115R	This study	
pLenti CMV hygro-FLAG-DNAJC5/CSPα L116Δ	This study	
pLenti CMV hygro-FLAG-DNAJC5/CSPα WT	This study	
pLenti CMV hygro-FLAG-DNAJC5/CSPα ΔLN	This study	
pLKO.1 sh-control	This study	
pLKO.1 sh-MmSLC3A2/CD98hc #1	This study	
pLKO.1 sh-MmSLC3A2/CD98hc #2	This study	
pPA-GFP C1-SLC3A2/CSPα WT	This study	
pPA-GFP C1-SLC3A2/CSPα ΔJ	This study	
pRK-FLAG-GFP(1–10)		[35]
**siRNAs**		
si-SLC3A2/CD98hc #1	Ambion	S12943
si-SLC3A2/CD98hc #2	Ambion	S12944
si-SLC3A2/CD98hc #3	Ambion	S12945
**Chemicals**		
Bafilomycin A_1_	Sigma	196,000
Cholera toxin subunit B (CF® 594 conjugated)	Biotium	00072
DAPI	Sigma	D9542
FN1 (fibronectin 1)	Sigma	F1141
3X FLAG® Peptide	Sigma	F4799
Hoechst 33,342, Trihydrochloride, Trihydrate	Thermo Fisher Scientific	H3570
Phalloidin (Alexa Fluor™ 594 conjugated)	Thermo Fisher Scientific	A12381
Streptavidin Agarose Resin	Thermo Fisher Scientific	20,349
Antibodies		
SNCA/α-Synuclein	BD bioscience	610,787
ANTI-FLAG® M2 Affinity Gel	Sigma	A2220
SLC3A2/CD98hc	Bethyl	A304-330A
SLC3A2/CD98hc	Thermo Fisher Scientific	PA5-96,401
DNAJC5/CSPα	Lsbio	LS-C22593
CLU (clusterin)	Santa Cruz Biotechnology	sc-5289
FLAG	Sigma	F1804
GFP	Santa Cruz Biotechnology	sc-9996
GFP-Trap Agarose	Chromotek	gta-20
HA	Sigma	H3663
HSPA8/HSC70	Santa Cruz Biotechnology	sc-7298
HSP90	Santa Cruz Biotechnology	sc-13,119
LAMP1	Santa Cruz Biotechnology	sc-18,821
mKeima	MBL	M182-3 M
Saposin A1	Xiaoyang Qi (University of Cincinnati College of Medicine)	
SEC61B/Sec61β		[35]
UBE1	Sigma	E3152
Mouse (Alexa Fluor™ 488 conjugated)	Thermo Fisher Scientific	A11029
Mouse (Alexa Fluor™ 568 conjugated)	Thermo Fisher Scientific	A11031
Rabbit (Alexa Fluor™ 488 conjugated)	Thermo Fisher Scientific	A11034
Rabbit (Alexa Fluor™ 568 conjugated)	Thermo Fisher Scientific	A11011
Mouse (HRP-conjugated)	Sigma	A4416
Rabbit (HRP-conjugated)	Sigma	A6154
Recombinant Protein G (HRP)	Abcam	ab7460
Mouse (Alexa Fluor™ 680 conjugated)	Thermo Fisher Scientific	A21058
Rabbit (IRDye800 conjugated)	Rockland	611–132-003

### Primary mouse neuron cultures

Primary cortical or hippocampal neuron cultures were prepared from P0-1 murine pups following an animal study protocol approved by NIDDK. Hippocampi were dissected in Hanks’ balanced salt solution (HBSS) and washed with MEM (Thermo, 11,095,080) twice. The hippocampi were incubated with 0.05% trypsin-EDTA (Thermo, 25,300,062) containing 100 µg/mL DNase-I (Sigma, 10,104,159,001) at 37°C for 10 min. Trypsin was then inactivated by addition of MEM containing 10% FBS. After washing with MEM three times, the tissues were dissociated by pipetting several times in MEM containing 100 µg/mL DNase-I. Cells were then centrifuged at 300 x g for 5 min and resuspended in the plating medium (MEM containing 10% FBS, 1 mM sodium pyruvate (Sigma, S8636), 2 mM L-glutamine (Sigma, 59202C), 100 µg/mL primocin (Invitrogen, ant-pm-05) and 0.6% glucose). The cell solution was passed through a 70-µm strainer (VWR, 76,327–100) once to filter out any cell clumps. Cells were seeded into plates or µ-slide 4 well chamber with glass bottom (ibidi, 80,427) pre-coated with 50 µg/ml poly-D-lysine (Sigma, P7280) and 2 µg/mL laminin (Sigma, L2020). Generally, we seed hippocampal cells obtained from a pup to cover 4 cm^2^ or 8 cm^2^ of surface area for Immunoblotting and imaging purpose, respectively. We seed cortical cells obtained from a pup to cover 15 cm^2^ surface area for immunoblotting. After incubation at 37 °C with 5% CO_2_ for overnight, the culture medium was changed to the Neurobasal media (Thermo, 21,103,049) containing 2% B27 (Thermo, 17,504,044), 0.5 mM L-glutamine and 100 µg/mL primocin to support the growth of hippocampal neurons. The culture media was replaced with fresh media once a week or when needed.

### Lentiviral infection

For neuron-specific expression of constructs, we replaced the inserted genes in Synapsin sHRPa-NRX in the FSW lentiviral vector (a gift from Alice Ting; Addgene, 73,147) with either *DNAJC5* or *SNCA*. For shRNA-mediated gene silencing of *Slc3a2*, we cloned annealed shRNA targeting sequences into pLKO.1 vectors (a gift from Bob Weinberg; Addgene, 8453) as listed below:

Scramble (control), forward primer: 5’-ccggCCTAAGGTTAAGTCGCCCTCGctcgagCGAGGGCGACTTAACCTTAGGtttttg-3’;

Scramble (control), reverse primer: 5’- aattcaaaaaCCTAAGGTTAAGTCGCCCTCGctcgagCGAGGGCGACTTAACCTTAGG-3’

Target 1, forward primer: 5’- ccggTGCAGCAAATTGTCAACATACctcgagGTATGTTGACAATTTGCTGCAtttttg-3’;

Target 1, reverse primer: 5’- aattcaaaaaTGCAGCAAATTGTCAACATACctcgagGTATGTTGACAATTTGCTGCA-3’;

Target 2, forward primer: 5’- ccggCCTGACCTCTTCTCCTACATActcgagTATGTAGGAGAAGAGGTCAGGtttttg-3’;

Target 2, reverse primer: 5’- aattcaaaaaCCTGACCTCTTCTCCTACATActcgagTATGTAGGAGAAGAGGTCAGG-3’

To prepare viruses, HEK293 FT cells (Thermo, R70007) in a 3.5-cm dish were transfected with 0.4 µg of pVSV-G (a gift from Bob Weinberg; Addgene, 8454), 0.6 µg of psPAX2 (a gift from Didier Trono; Addgene, 12,260), and 0.8 µg of lentiviral expression vectors for 24 h and the media was replaced with 3 mL of fresh media for 48 h to collect viruses. After harvesting viral soups and centrifugation at 1,000 xg for 10 min, the soups were filtered through 0.45 µm PVDF syringe filter unit (Sigma, SLHVM33RS) and stored in −80°C until usage. Neurons (12 well plate) were infected with 200 µl of viral soups together with 200 µl of EGFP-human SNCA viruses in 1 mL total volume of media at DIV3 (days in vitro). The infecting media was replaced with fresh media at DIV4 and further incubated for one week before the assays.

### Protein samples preparation and Immunoblotting

Conditioned media and cell lysates were prepared as described previously [19,35]. Briefly, cells (2.5 × 10^5^) seeded in a poly-D-lysine-coated 12-well plate were grown for 24 h, and then transfected with 250 ng plasmids expressing the indicated MAPS substrates together with 250 ng plasmids expressing a MAPS regulator (e.g., DNAJC5). We replaced the medium with 1.5 mL fresh DMEM medium 24 h post-transfection. Cells were grown for another 16 h before conditioned media were collected. The media were subjected to sequential centrifugation, first at 1000 × g for 5 min to remove contaminated cells, and then at 10,000 × g for 30 min to remove cell debris. Cells were lysed in 200 μl NP40 lysis buffer (0.5% Nonidet P-40 [Sigma, 56,741–250ML-F], 50 mM Tris-HCl, pH 7.4, 150 mM NaCl, 2 mM MgCl_2_, 1 mM EDTA, 1 mM DTT, protease inhibitors [Sigma, 11,697,498,001]. A fraction of the cell lysates and media were analyzed by SDS–PAGE and immunoblotting. Unless specified in figurelegends, protein secretion is normalized by the expressed protein in cell lysates. Immunoblottings were performed using the standard protocols. All primary antibodies were diluted in 5% BSA (Sigma, A9418) in phosphate-buffered saline (PBS; Corning, 21–040) as described in Table 1. To quantify secreted protein in conditioned media, HRP-conjμated secondary antibodies were used. Immunoblotting signal was detected by the enhanced chemiluminescence method (ECL) and recorded by a Fuji LAS-4000 imager or Bio-Rad Chemidoc. The intensity of the detected protein bands was quantified by ImageGauge v3.0, Bio-Rad ImageLab, or ImageJ. Protein secretion levels were determined by normalizing the level of the secreted proteins by the amount of the same protein in cell lysates. For other immunoblotting quantifications, fluorescently labeled secondary antibodies were used. Immunoblots were scanned by a LI-COR Odyssey scanner or Bio-Rad Chemidoc.

### Immunoprecipitation

For co-immunoprecipitation assays, pre-equilibrated FLAG M2 agarose beads (Sigma, A2220) or GFP-Trap beads (Chromotek, gta-20) were incubated with lysates containing tagged proteins for 1 h at 4°C. The beads were washed with the lysis buffer, and the bound proteins were eluted in 1x Laemmli buffer at 95°C and resolved by SDS-PAGE for immunoblotting analysis. For co-IP of endogenous DNAJC5 with SLC3A2 from neurons, lysates of cortical neurons from 3 wells of a 6 well plate were combined. Cell lysates were prepared in 1.5 mL volume using either CHAPS lysis buffer (1% CHAPS [Sigma, 10,810,118,001], 50 mM HEPES, pH 7.4, 100 mM NaCl, 1 mM DTT, protease inhibitors) or NP40 lysis buffer. The lysates were pre-cleared with 75 ul of protein A agarose (Sigma, IP02) equilibrated with 3% BSA, for 4 h at 4°C. After centrifugation at 2,500 x g for 2 min, 45 ul of lysates was saved as input and the remaining samples were used for immunoprecipitation with 2.5 µg of rabbit IgG or SLC3A2 antibody (Thermo, PA596401) overnight at 4°C. The immunocomplexes were affinity isolated using 20 μL of pre-equilibrated protein A beads for 2 h at 4°C, followed by washing in the same lysis buffer. The immunoprecipitates were eluted by 1 x Laemmli sample buffer, heat-denatured at 95°C for 5 min before SDS-PAGE electrophoresis and immunoblotting. To avoid IgG bands on the IP blot, protein G-HRP (abcam) was used as secondary antibody.

### Tandem affinity purification and mass spectrometry of FLAG-SBP-DNAJC5 precipitates

A schematic flow chart of the purification procedure is presented in Figure S3A. Three p150 dishes of HEK293T cells, which were transfected with 15 μg of either empty vector, FLAG-Streptavidin binding peptide (SBP)-tagged *DNAJC5 WT* or *DNAJC5 ΔJ*, were harvested in PBS. After centrifugation at 1,000 x g for 10 min at 4°C, the cell pellets were treated with 0.025% digitonin (Sigma, D141) in the PB buffer (230 mM potassium acetate [Sigma, P1190], 10 mM sodium acetate [Sigma, S2889], 50 mM HEPES, pH7.3, 5 mM MgCl_2_, 1 mM EGTA [Sigma, 324,626]) containing 1 mM DTT and a protease inhibitor cocktail for 5 min. Once membrane permeabilization is confirmed by trypan blue staining, the lysates were spun at 16,000 x g for 5 min. The resulting membrane pellets were washed with 1x PB buffer followed by centrifugation. The washed pellets were resuspended in 0.33% formaldehyde in PB buffer and incubated at 37°C for 25 min with rocking for crosslinking. After top-speed centrifugation for 5 min, the pellets were further lysed in 8 mL of RIPA lysis buffer (50 mM Tris pH7.5, 1% Nonidet P-40, 0.1% SDS, 2 mM EDTA pH8.0, 0.5% sodium deoxycholate [Sigma, D6750], 150 mM NaCl) with 1 mM DTT and a protease inhibitor cocktail for 1 h at 4°C. After centrifugation at 16,000 x g for 5 min, the supernatants were collected for the following tandem affinity purification steps.

To purify DNAJC5 and its interacting proteins, the RIPA-soluble fractions were incubated with 200 μl of pre-washed FLAG M2 agarose bead slurry with rocking for 1 h at 4°C. The beads were washed with 1 mL of RIPA buffer three times and 1 mL of streptavidin binding buffer (10 mM Tris-HCl, pH 7.5, 1 mM EDTA, 1 M NaCl) once. After spinning down at 500 x g for 5 min, the bound proteins were eluted by incubation with 250 μl of 3 x FLAG peptide solution (200 μg/mL; Sigma, F4799) for 15–20 min twice. After centrifugation, the eluates (~500 μl) were incubated with an equal volume of streptavidin binding buffer and 80 μl of pre-washed streptavidin beads (Thermo, 20,349). After incubation for 1 h at 4°C, the beads were washed with streptavidin binding buffer three times. The bound proteins were eluted in 40 μl 1x Laemmli buffer by boiling for 20 min. The purified proteins were resolved by SDS-PAGE and visualized by silver staining using the SilverQuest kit (Invitrogen, LC6070) according to the manufacturer<apos;>s protocol. Mass spectrometry analysis of DNAJC5 co-purified proteins in gel slices was done by the Taplin Mass Spectrometry facility of Harvard Medical School by a fee-based service. Briefly, excised gel bands were subjected in-gel trypsin digestion using 12.5 ng/µl modified sequencing-grade trypsin (Promega, PRV5111) at 4°C for 45 min. After digestion, the excess trypsin solution was removed and replaced with 50 mM ammonium bicarbonate solution to just cover the gel pieces. Samples were then placed in a 37°C room overnight. Peptides were later extracted by removing the ammonium bicarbonate solution, followed by one wash with a solution containing 50% acetonitrile and 1% formic acid. The extracts were then dried in a speed-vac. The dried samples were reconstituted in 5–10 µl of HPLC solvent A (2.5% acetonitrile, 0.1% formic acid). A nano-scale reverse-phase HPLC capillary column was created by packing 2.6 µm C18 spherical silica beads into a fused silica capillary (100-µm inner diameter x ~ 30-cm length) with a flame-drawn tip. After equilibrating the column each sample was loaded via a Famos auto sampler (LC Packings, 920) onto the column. A gradient was formed and peptides were eluted with increasing concentrations of solvent B (97.5% acetonitrile, 0.1% formic acid). Eluted peptides were subjected to electrospray ionization and then injected into an LTQ Orbitrap Velos Pro ion-trap mass spectrometer (Thermo, FSN05-10,001). Peptides were detected, isolated, and fragmented to produce a tandem mass spectrum of specific fragment ions for each peptide. Peptide sequences (and hence protein identity) were determined by matching protein databases with the acquired fragmentation pattern by the software program, Sequest (Thermo). All databases include a reversed version of all the sequences and the data was filtered to between a one and two percent peptide false discovery rate (FDR).

### Imaging analysis

For immunostaining, cells cultured on #1.5 thickness coverslips precoated with poly-D-lysine (50 μg/mL; Thermo, A3890401) were fixed in 4% paraformaldehyde in PBS or in cold methanol for 10 min. Cells were then washed with PBS twice and permeabilized with a PBS-based staining solution containing 0.2% saponin (Sigma, 47,036) and 10% FBS for 10 min at room temperature. Cells were stained by primary antibodies diluted in the staining solution overnight at 4°C and washed with PBS three times. Alexa Fluor® secondary antibodies (Thermo; see Table 1) were diluted in the staining solution and added to cells for 1 h at room temperature. If necessary, 1 μg/mL DAPI (Sigma, D9542) was included in the staining solution to label nuclei. After washing with PBS three times, the coverslips were mounted on glass slides with mounting media (Vectashield, H-1000-10). For live-cell imaging, cells were seeded to μ-slide chamber (ibidi, 80,426) precoated with 10 μg/mL FN1 (fibronectin 1; Sigma, F0556) for 1 h. Culture media were replaced with imaging medium (phenol-red free DMEM containing 10% FBS) before imaging. Cells were stained with LysoTracker Red (Thermo, L7528) or CF^594^-labeled cholera toxin subunit B (CTB; Biotium, 00072) according to the manufacturer<apos;>s protocols. AFSM images were detected using a DAPI filter (Ex_405_/Em_427-487_). Photobleaching experiments were performed as described previously [35]. Cell imaging experiments were conducted on either a LSM780 laser scanning confocal microscopy (Zeiss) or a SoRa spinning disk super-resolution microscopy (Nikon) equipped with heating and a CO_2_ incubation system. Images were further processed for dot number counting or fluorescence ratio determination using ImageJ. For colocalization assay, images were denoised using Nikon NIS-element plugin Denoise.ai and the colocalization tools in the NIS-elements software (Nikon), colocalization tools in the Zeiss Zen (black) software, or JACoP plugin in ImageJ were used.

### Flow cytometry

*Keima*-expressing cells were dissociated into fresh DMEM medium by gentle pipetting and passed through a cell strainer cap filter (Thermo, 08–771-23). Flow cytometry was performed on an LSRII Fortessa analyzer (Becton Dickinson). The gate for acidic (Ex_586_/Em_620_)/neutral (Ex_440_/Em_620_) intensities of individual cells (>10,000 cells) were determined manually by reference of bafilomycin A_1_ (100–200 nM for 2–4 h) treated samples, which converts ~99% of cell population to the neutral gate. Flow data were analyzed using FlowJo 10.6 software (FlowJo LLC).

### Fly experiments

Fly strains bearing shRNA-expressing cassettes downstream of UAS sequences are 31,603, 35,785, 57,746 from the Bloomington Drosophila Stock Center. The flies expressing either *WT human DNAJC5* or *DNAJC5^L116Δ^* were described previously [41]. Unless otherwise specified, cultures were maintained in 25°C incubators on BDSC cornmeal food (Homemade based on the recipe from Genesee Scientific). For imaging fly eyes, 5–10 adult flies were fixed in PBS containing 4% formaldehyde for 1 h, rinsed with PBS. The flies were then dehydrated by soaking sequentially in 30%, 50%, 70%, 90%, and 100% ethanol. Dried flies were mounted in an Ibidi imaging chamber and scanned by a Nikon C1 spinning disk confocal microscope using Ex_488_/Em_520_ nm. Shown is the maximum projection view of the scanned Z-section images.

Two methods were used to score the eye phenotypes, which generate similar results. One analysis done in the Zinsmaier lab was as described previously [41]. Briefly, eye phenotypes of 1 – to 4-day-old adults were digitally imaged using a Nikon stereomicroscope. A semi-quantitative assessment of the eye phenotypes was achieved by serially coding the obtained images and blind scoring using naïve researchers. Eye phenotypes were given a relative score in comparison to known scoring classes: (0) normal WT-like eye. (1) Mild: slightly ‘rough’ eye surface and/or slight dis-colorization; (3) Moderate: significant dis-colorization and rough eye surface, slightly disorganized ommatidia and/or reduced size; (5) Severe: Discolored and deformed eye due to loss of or malformed ommatidia, significant reduction in eye size. Scores were collected for at least 5 individual flies per genotype derived from at least 4 individual crosses. Alternatively, eye images were processed by ImageJ to measure the size of the area that have lost the eye pigments.

To detect AFSM in photoreceptor cells, imaginal eye discs were dissected from third instar larvae, fixed in 4% formaldehyde in PBS for 20 min at room temperature. Eye discs were washed three times with PBS, then permeabilized in a PBS-based staining solution containing 0.2% saponin and 10% FBS for 10 min. The discs were then stained by Alexa Fluor 594-labeled phalloidin (Thermo, A12381) in the staining solution for 30 min at room temperature. The discs were washed twice by PBS and then imaged by a Zeiss LSM780 laser scanning confocal microscope.

### Quantitative RT-PCS (qRT-PCR)

Total RNA extracts were obtained from primary mouse neurons or 3^rd^ instar larvae, using RNeasy Mini Kits (Qiagen, 74,004) according to the manufacturer<apos;>s protocol. The cDNAs were synthesized by iScript™ Reverse Transcription Supermix (Bio-Rad, 1,708,840) and were then subject to real time qPCR analyses using SsoAdvanced Universal SYBR Green Supermix (Bio-Rad, 1,725,270) with following primers:

*mSlc3a2*-F: 5’-CGACCTTCAGGCCTTTGTAG-3’

*mSlc3a2*-R: 5’-CTAACACCAGGCCCTTCAC-3’

*mβ-Actb*-F: 5’-CATTGCTGACAGGATGCAGAAGG-3’

*mβ-Actb*-R: 5’-TGCTGGAAGGTGGACAGTGAGG-3’

*dSlc3a2*-F: 5’-GCAGCCACTTCCATGGTACA-3’

*dSlc3a2*-R: 5’-TAAATCGCGCCAGCGGAAA-3’

*dRp49-F*: 5’-AGATCGTGAAGAAGCGCACCAAG-3’

*dRp49-R*:5’-CACCAGGAACTTCTTGAATCCGG-3’

### Statistical analysis

All experiments were repeated two or more times. For statistical analyses, at least three independent experiments were carried out. For quantification of microscope images, at least 30 randomly selected cells or at least 10 randomly selected fields were analyzed. Statistical analysis for experiments with two treatment groups used one or two-tailed t-test. For more than two groups, we used one-way ANOVA followed by multiple comparison analyses of variance by the Dunnett<apos;>s test where all groups were compared back to a single control group. Tukey<apos;>s multiple tests were used where all groups were compared. Differences were considered significant at the 95% level of confidence. Details of statistical tests used, number of biological replicates (n), and *P* values for each experiment are included in figure legends. All statistical analyses and graphing were performed using GraphPad Prism 9.

## Supplementary Material

Supplemental Material

## Data and materials availability

All the data needed to evaluate the conclusions in the paper are present in the paper and/or the Supplementary Materials. Additional data related to this paper may be requested from the authors.
