# Supplementary material for: Abnormal triaging of misfolded proteins by adult neuronal ceroid lipofuscinosis-associated DNAJC5/CSPα mutants causes lipofuscin accumulation
Source: Autophagy. 2022 May 4;19(1):204–23. doi: 10.1080/15548627.2022.2065618 (PMC9809949; doi:10.1080/15548627.2022.2065618)
Supplement: Supplemental Material [file KAUP_A_2065618_SM0013.zip › Supplementary_materials_revised_0075R1_clean R3_JL.docx]

**Supplementary Figures**

**
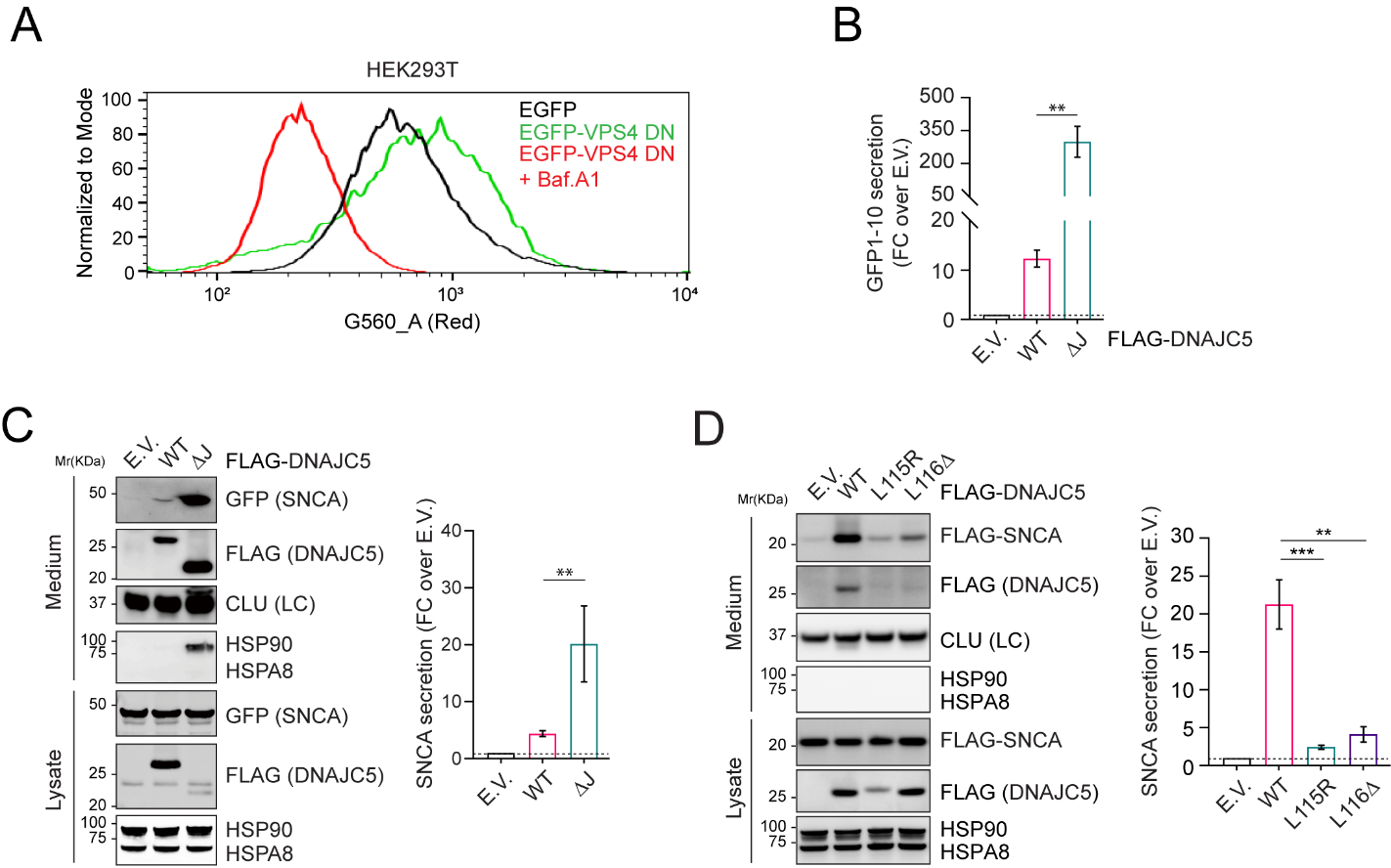
**

**Figure S1.** DNAJC5-mediated microautophagy is dispensable for MAPS. (**A**) VPS4 DN expression does not increase the lysosomal pH. HEK293T cells transfected with *EGFP-VPS4 DN^E228Q^* were stained with a LysoTracker dye and subjected to flow cytometry analysis. Two gates were selected for comparison of EGFP-negative and -positive cells. Bafilomycin A_1_ (200 nM for 2 h) treatment was used as the positive control. (**B**) Quantification of the experiments as shown in Fig. 2G; error bars, mean ± s.e.m. for fold changes in the ratio of GFP1-10_Med_:GFP1-10_Lys.._ relative to E.V. control (dotted line), from at least 3 independent experiments. **, *p* < 0.01 *(p* = 0.0044) by two-tailed Student t-test. (**C**) Secretion of EGFP-SNCA is stimulated by DNAJC5 WT and DNAJC5 ΔJ. HEK293T cells expressing *GFP-SNCA* were transfected with empty vector (EV) or *DNAJC5* plasmids. Secreted proteins were collected for 16 h and analyzed by immunoblotting as indicated. The graph shows mean ± s.e.m. for fold changes in the ratio of SNCA_Med_:SNCA_Lys_ relative to E.V. control (dotted line), from at least 3 independent experiments. **, *p* < 0.01 *(p* = 0.0047) by two-tailed t-test. Note that a fraction of HSP90 but not HSPA8 was released when SNCA and DNAJC5 ΔJ were co-expressed. (**D**) DNAJC5 ANCL mutants fail to induce SNCA secretion. HEK293T cells expressing *FLAG-SNCA* were transfected with the indicated *DNAJC5* plasmids. Conditioned medium (16 h) and cell lysates were analyzed by immunoblotting. The graph shows mean ± s.e.m. for fold changes in the ratio of SNCA_Med_:SNCA_Lys_ relative to E.V. control (dotted line), from *n* = 3 independent experiments. ***, *p* < 0.0005 (*p* = 0.0010); **, *p* < 0.01 (*p* = 0.0016) by one-way ANOVA plus Dunnett’s multiple comparisons test. LC, loading control. Related to Fig. 2.

**
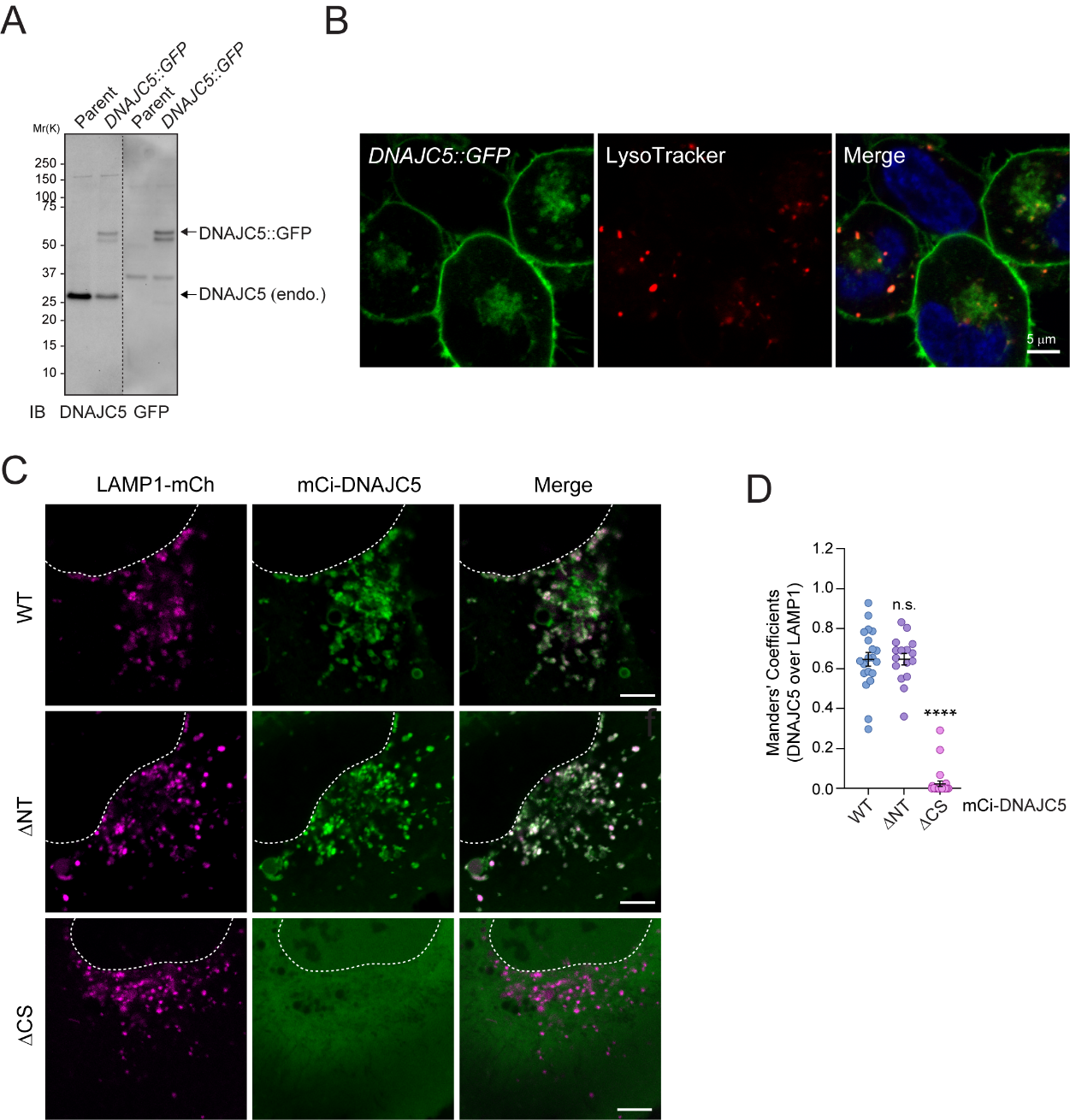
**

**Figure S2.** The linker domain localizes DNAJC5 to a perinuclear LAMP1-negative compartment and is required for MAPS. (**A**) Validation of endogenous tagging in *DNAJC5::GFP* HEK293T cell. Lysates from the indicated cells were analyzed by immunoblotting using antibodies against DNAJC5 and GFP. (**B**) DNAJC5 is partially colocalized with the LysoTracker-labeled lysosomes. *DNAJC5::GFP* HEK293T cells were stained with LysoTracker dye (1:10,000 for 10 min at 37°C) and imaged by live cell imaging. Nuclei were stained with Hoechst 33342. (**C** and **D**) Colocalization of DNAJC5 ΔNT and ΔCS with LAMP1-mCh. (C) Representative confocal images of U2OS cells transfected with *LAMP1-mCh* together with the indicated *mCitrine-tagged DNAJC5* variants. Scale bars: 5 μm. (D) Quantification of the colocalization efficiency of various DNAJC5 variants with LAMP1-mCh in individual cells in (D). Error bars indicate mean ± s.e.m. ****, *p* < 0.0001 by one-way ANOVA plus Dunnett’s multiple comparisons test; n.s, not significant; *n* = 20, 16 and 25 cells respectively from 3 independent experiments. Related to Fig. 4.

**
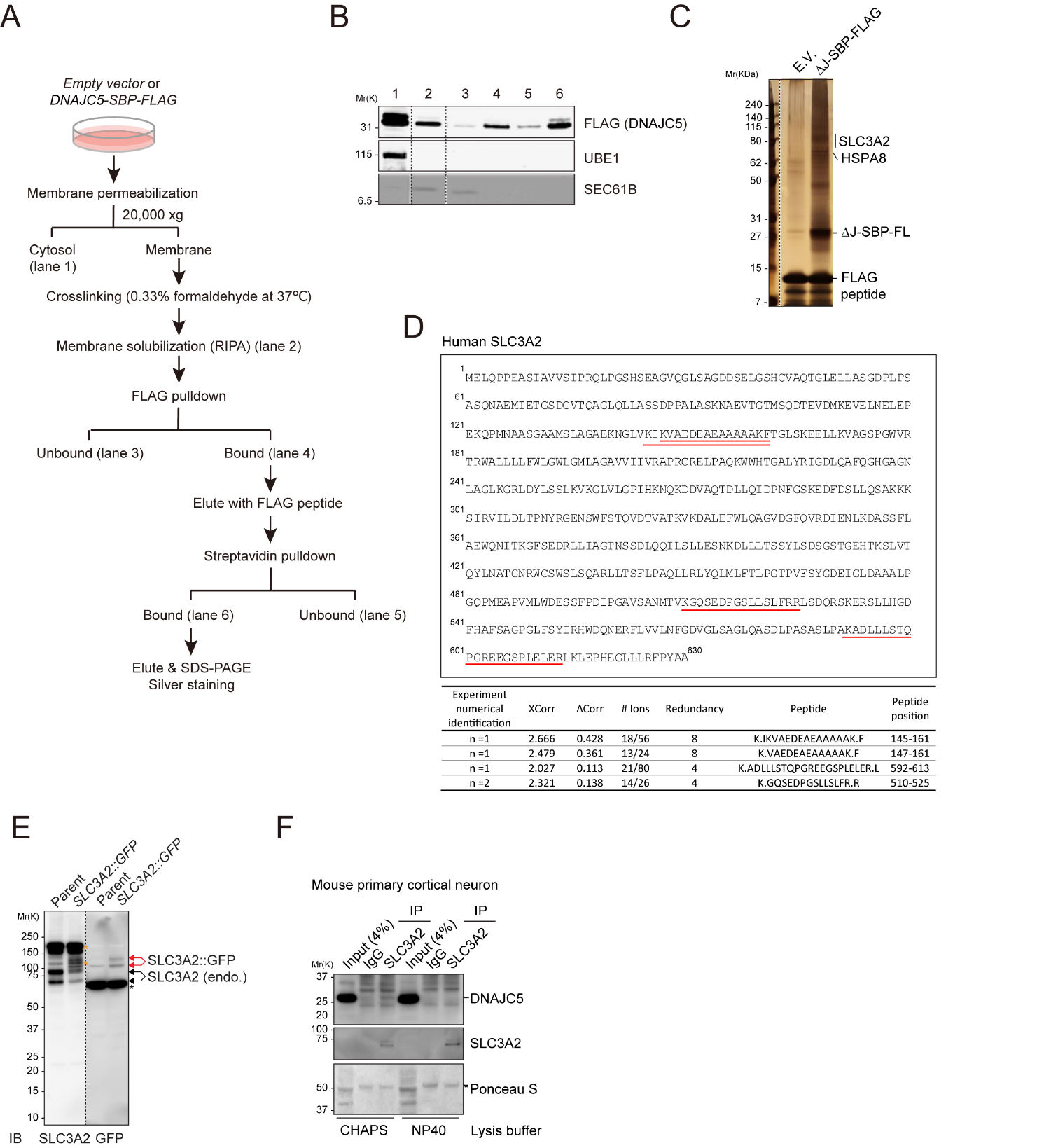
**

**Figure S3.** SLC3A2 interacts with DNAJC5 via the LN domain and is required for the perinuclear localization of DNAJC5. (**A**) A schematic flow chart for crosslinking and tandem affinity purification to identify DNAJC5 ΔJ-SBP-FLAG-associated proteins. See the method section for details. (**B**) Sample from the steps indicated in (A) were subjected to immunoblotting to verify the fractionation and purification procedure. (**C**) A silver-stained gel showing the proteins co-purified with DNAJC5 ΔJ-SBP-FLAG from a second experiment. (**D**) The peptide fragments identified by LC-MS/MS are underlined (red) on human SLC3A2 sequences (UniProtKB - P08195). Briefly, bands between 70~100 kDa on the silver-stained gels in Fig. 5A (n =1) and Fig. S3C (n = 2) were sectioned and analyzed by LC-MS/MS. SLC3A2 was one of proteins with unique peptide hits not found in the empty vector control sample from the two independent experiments. The table summarizes the mass spectrometry data for SLC3A2-derived peptides identified from the two independent experiments. (**E**) Validation of endogenous tagging in *SLC3A2::GFP* HEK293T cell. Lysates from the indicated cells were analyzed by immunoblotting using SLC3A2 and GFP antibodies. Note the two upper bands indicated by the red arrows are detected by both antibodies at the same size. (**F**) Interaction between endogenous SLC3A2 and DNAJC5 in neurons. Mouse primary cortical neurons (DIV10) were lysed with either CHAPS or NP40 lysis buffer, followed by immunoprecipitation using either SLC3A2 antibody or rabbit IgG. The lysate input and immunoprecipitates were analyzed by immunoblotting as indicated. Note that SLC3A2-associated DNAJC5 was only detected under CHAPS lysis condition. The asterisk in the ponceau S-stained membrane indicates IgG bands. Related to Fig. 5.

**
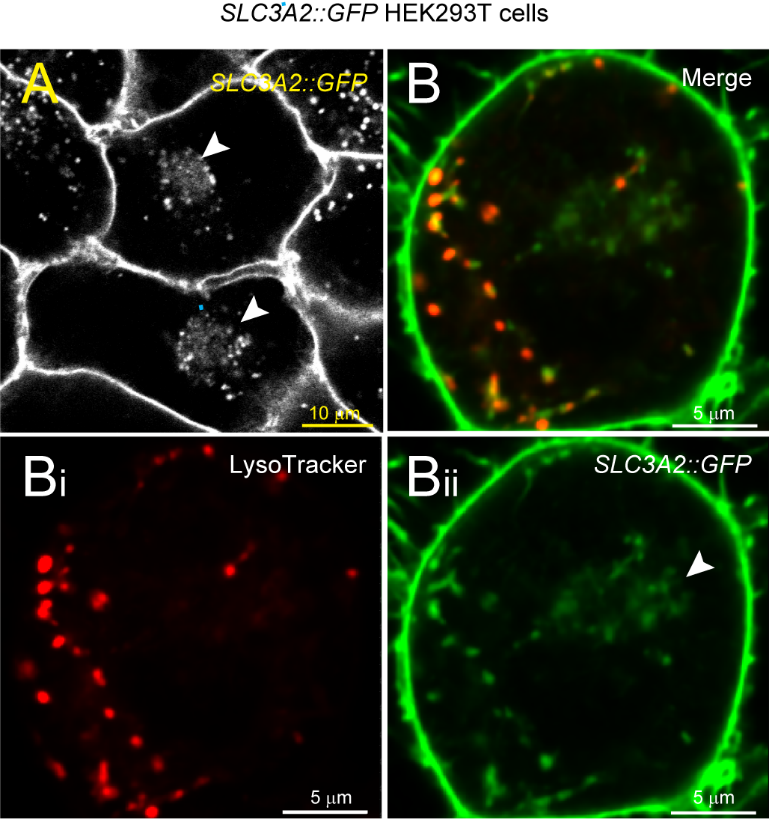
**

**Figure S4.** SLC3A2 interacts with DNAJC5 via the LN domain and is required for the perinuclear localization of DNAJC5. (**A**) HEK293T cells expressing *SLC3A2* bearing an endogenously tagged GFP were imaged by live cell confocal microscopy. (**B**) The cells were stained a LysoTracker dye in red for 10 min and imaged by live cell confocal microscopy. Arrowheads indicate perinuclear SLC3A2 clusters barely labeled by the LysoTracker dye. Related to Fig. 5.

**
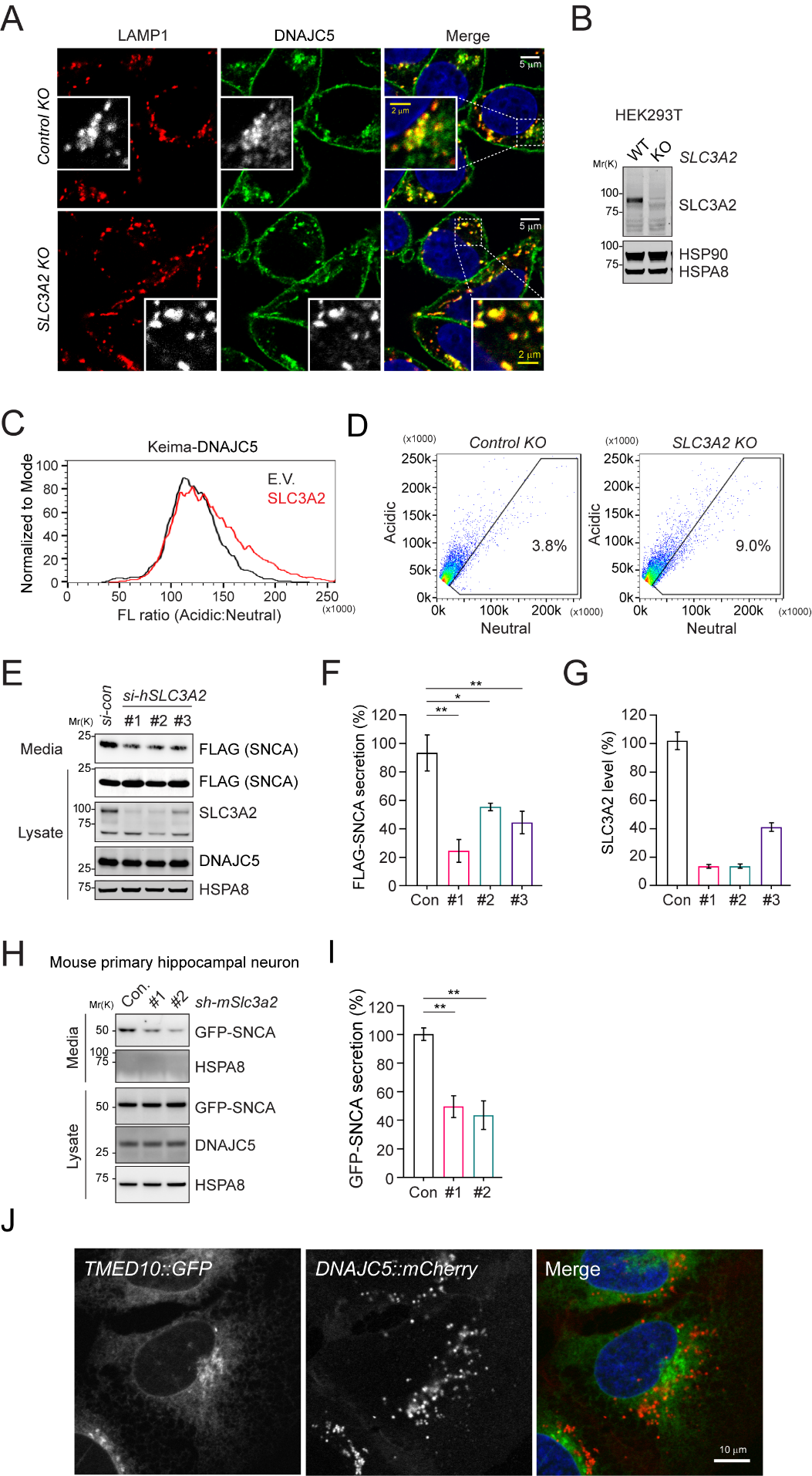
**

**Figure S5.** Depletion of SLC3A2 reduces DNAJC5 localization to the perinuclear LAMP1 negative compartment and inhibits MAPS. (**A**) *SLC3A2* CRISPR KO or control KO HEK293T cells were double stained for endogenous LAMP1 (red) and DNAJC5 (green) and imaged by confocal microscopy. DAPI (in blue) was used to label the nuclei. The insets show an enlarged view of the box-indicated area. (**B**) The knockout of *SLC3A2* was confirmed by immunoblotting using cell lysates prepared from a fraction of cells in (A). (**C**) Overexpression of *SLC3A2* only slightly enhance the translocation of DNAJC5 into endolysosomes. Keima-DNAJC5 expressing HEK293T cells were transfected with either an empty vector or *SLC3A2-Myc-FLAG*-expressing vector and analyzed by flow cytometry. The graph shows the acidic/neutral fluorescence ratio from > 10,000 cells of the Keima-positive population. (**D**) Depletion of *SLC3A2* did not significantly affect the translocation of DNAJC5 into endolysosomes. Control KO and *SLC3A2* KO HEK293T cells were transiently transfected with Keima-DNAJC5 and analyzed by flow cytometry. Dot plots show a selected neutral gate, indicating that most Keima-DNAJC5 is still translocated into endolysosomes in *SLC3A2* KO cells. (**E**) SLC3A2 is required for SNCA secretion. HEK293T cells were transfected with either control siRNA or three different siRNA targeting *SLC3A2*. 24 h post transfection, cells were re-seeded and further transfected with *FLAG-human SNCA*. The conditioned medium (16 h) and lysates were analyzed by immunoblotting as indicated. (**F**) Quantification of the SNCA secretion from the experiments in (E). Error bars, mean ± s.e.m. (*n* = 3); **, *p* < 0.01 (p = 0.0012 and 0.0095, respectively; *, *p* < 0.05 (p = 0.0344) by one-way ANOVA plus Dunnett’s multiple comparisons test. (**G**) Quantification of the SLC3A2 levels from the experiments in (E) (normalized by HSPA8 level). error bars, mean ± s.e.m., *n* = 3 independent experiments. (**H** and **I**) SLC3A2 is required for SNCA secretion in neuron. Mouse primary hippocampal neurons were infected with lentiviruses expressing GFP-tagged human *SNCA* and the indicated shRNAs at DIV3. The conditioned medium (16 h) and lysates obtained at DIV11~12 were analyzed by immunoblotting as indicated. I, Quantification of the experiments in (H) (*n* = 3). Error bars indicate mean ± s.e.m.; **, *p* < 0.01 (*p* = 0.006 and 0.0035, respectively) by one-way ANOVA plus Dunnett’s multiple comparisons test. (**J**) The DNAJC5 positive perinuclear compartments are not significantly enriched with TMED10. *DNAJC5::GFP* U2OS cells were engineered to express *TMED10* bearing an endogenously tagged mCherry at the carboxyl terminus. The cells were analyzed by live cell confocal microscopy. Nuclei were stained with Hoechst 33342. Related to Fig. 5 and 6.

**
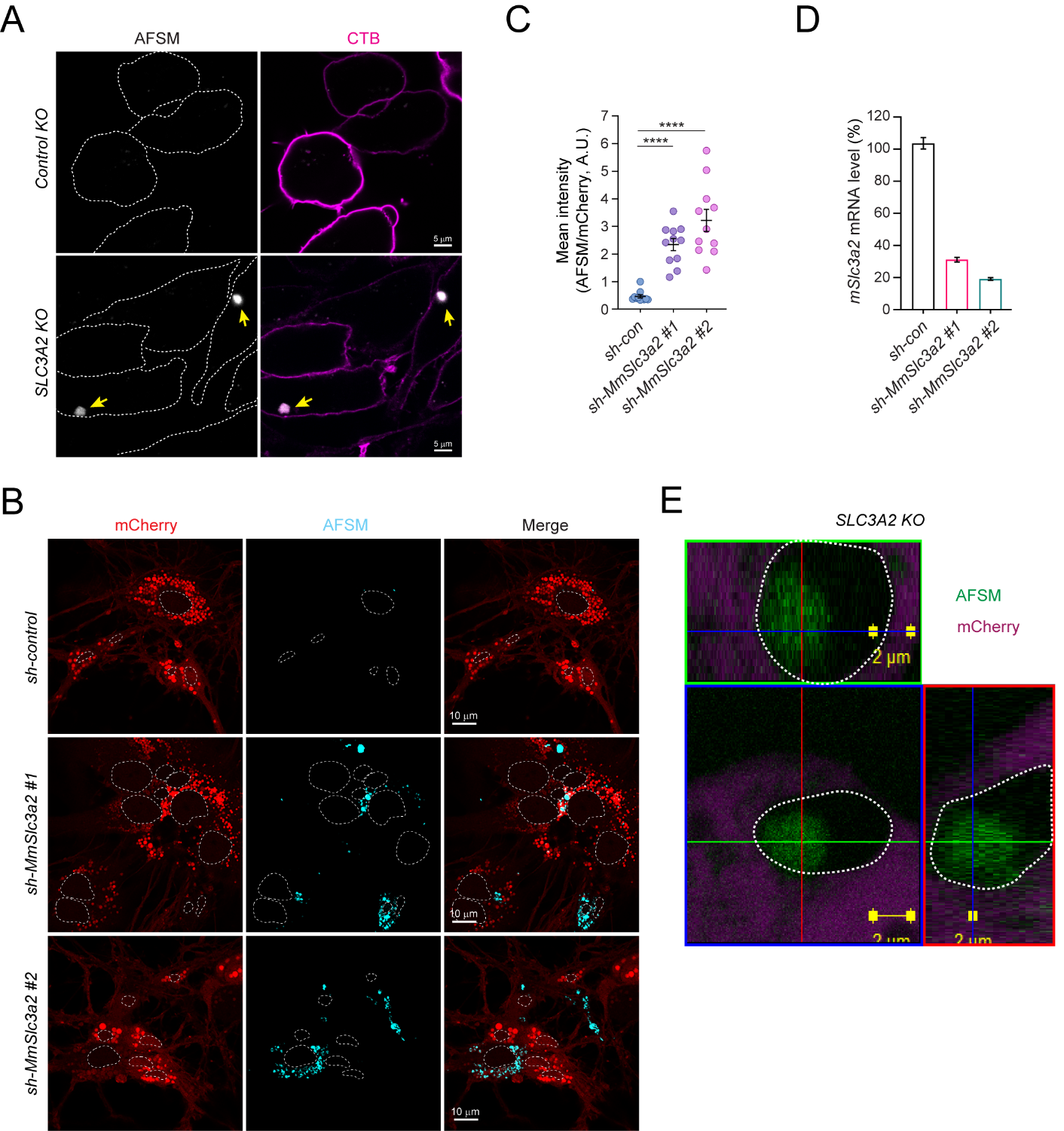
**

**Figure S6.** Depletion of SLC3A2 causes accumulation of AFSMs in cells. (**A**) Control KO or *SLC3A2* KO HEK293T cells were treated with cholera toxin subunit B (CTB) to label cell boundary. Cells were imaged by confocal microscopy. Arrows indicate AFSMs. Dashed lines show cell boundaries. (**B** and **C**) shRNA knockdown of *MmSlc3a2* induces AFSM in neurons. Mouse primary hippocampal neurons (DIV3) were infected with lentivirus expressing either *MmSlc3a2* or control shRNA, together with *mCherry* driven by the *Syn1* promoter to label neurons. The neurons were imaged at DIV10 by confocal microscopy. The dot lines indicate nuclei. Scale bars: 10 µm. (C) Quantification of the mean intensities ± s.e.m of AFSM in (B), normalized by mCherry signals from randomly selected fields (*n* = 11). Error bars indicate mean ± s.e.m. ****, *p* < 0.0001 by one-way ANOVA plus Dunnett’s multiple comparisons test.

(**D**) qRT-PCR confirms the knockdown of *MmSlc3a2* mRNA in neurons used in Fig. S5H and S6B. Total RNAs were extracted from a fraction of the neurons cultured and infected in parallel. Quantitative real-time PCR was then used to determine the relative *MmSlc3a2* mRNA levels (normalized by *MmActb* mRNA level). (**E**) Orthogonal views (x/y, x/z or y/z) of confocal z-stacks of a *SLC3A2* KO cell transfected with mCherry (magenta). Note that the AFSM puncta (green) is localized in a cavity in the cytoplasm. Related to Fig. 6.

**
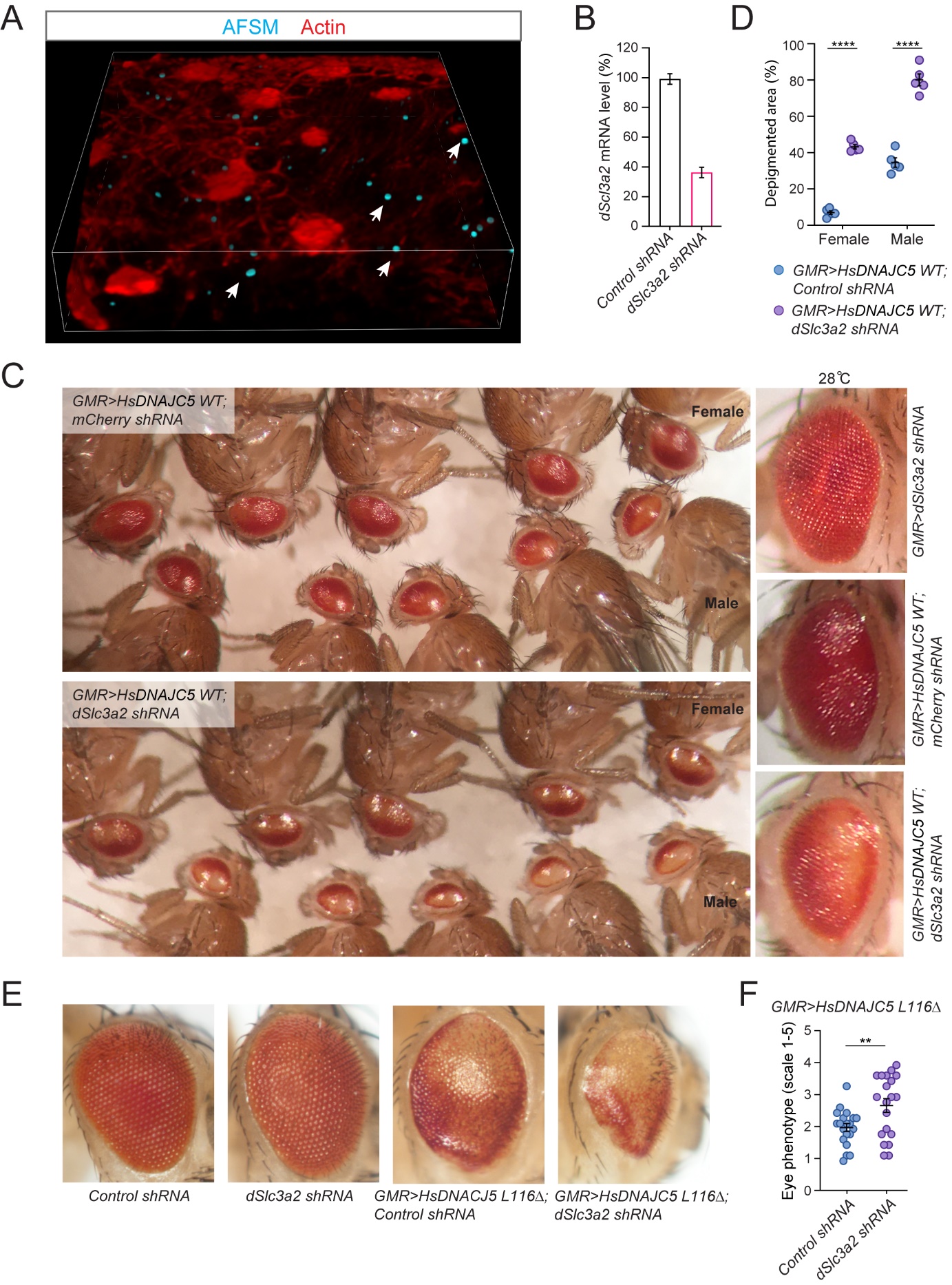
**

**Figure S7.** AFSM accumulation and genetic interaction between DNAJC5 and SLC3A2 in a fly ANCL model. (**A**) Accumulation of AFSMs in photoreceptor cells in an eye disc from a *GMR>HsDNAJC5*^L116∆^ larva. Shown is a 3-D view reconstructed from confocal z-stacks of an eye disc stained by phalloidin. Arrows indicate examples of AFSMs. (**B**) Knockdown of *dSlc3a2* mRNA by the fly line used in Figure 7 and fig. S7. Total RNAs were extracted from 3^rd^ instar larvae of the following genotypes (*hs-gal4/UAS-sh-mCherry* or *hs-gal4/UAS-sh-dSlc3a2*) after 5 heat shocks at 37°C for 30 min. cDNAs were synthesized and analyzed by qRT-PCR to determine relative *dSlc3a2* mRNA levels normalized by *dRp49* mRNA level. (**C**) *dSlc3a2* knockdown enhances the rough eye and depigmentation phenotypes in *GMR>hDNAJC5 WT* flies at 28°C. Five representative females and males are imaged. The right panels are enlarged images of female fly eyes of the indicated genotypes. (**D**) Quantification of the depigmented area of fly eyes. Images in (C) were analyzed using the Fiji software to measure the size of depigmented eye areas. Error bars indicate mean ± s.e.m.; ****, *p* < 0.0001; **, *p* < 0.01 (*p* = 0.0016) by two-tailed t-test in each female and male groups. (**E** and **F**) *dSlc3a2* knockdown enhances the rough eye phenotype induced by hDNAJC5^L116Δ^ at 25°C. (E) Representative images of fly eyes of the indicated genotypes. (F) Quantification of the eye phenotypes in (E) (see methods). Scores were collected for at least 5 individual flies per genotype derived from at least 4 individual crosses. Error bars indicate mean ± s.e.m.; **, *p* < 0.01 (*p* = 0.0085) by two-tailed t-test. Related to Fig. 7.

**Movie S1.** Colocalization of endogenous DNAJC5 and SLC3A2. HEK293 cells bearing GFP and mCherry tag on DNAJC5 and SLC3A2 respectively were imaged by dual-color live cell confocal microscopy. Hoechst 33342 was used for staining of nuclei.

**Movie S2.** Colocalization of endogenous DNAJC5 and LysoTracker-positive compartments. *DNAJC5::GFP* HEK293T cells were stained with a LysoTracker dye (1:10,000) and imaged in real time by a confocal microscope. Hoechst 33342 was used for staining of nuclei.
